# Constraints-based analysis identifies NAD^+^ recycling through metabolic reprogramming in antibiotic resistant *Chromobacterium violaceum*

**DOI:** 10.1371/journal.pone.0210008

**Published:** 2019-01-04

**Authors:** Deepanwita Banerjee, Anu Raghunathan

**Affiliations:** Chemical Engineering Division, CSIR-National Chemical Laboratory, Pune, Maharashtra, India; University of Nebraska Medical Center, UNITED STATES

## Abstract

In the post genomic era, high throughput data augment stoichiometric flux balance models to compute accurate metabolic flux states, growth and energy phenotypes. Investigating altered metabolism in the context of evolved resistant genotypes potentially provide simple strategies to overcome drug resistance and induce susceptibility to existing antibiotics. A genome-scale metabolic model (GSMM) for *Chromobacterium violaceum*, an opportunistic human pathogen, was reconstructed using legacy data. Experimental constraints were used to represent antibiotic susceptible and resistant populations. Model predictions were validated using growth and respiration data successfully. Differential flux distribution and metabolic reprogramming were identified as a response to antibiotics, chloramphenicol and streptomycin. Streptomycin resistant populations (StrpR) redirected tricarboxylic acid (TCA) cycle flux through the glyoxylate shunt. Chloramphenicol resistant populations (ChlR) resorted to overflow metabolism producing acetate and formate. This switch to fermentative metabolism is potentially through excess reducing equivalents and increased NADH/NAD ratios. Reduced proton gradients and changed Proton Motive Force (PMF) induced by antibiotics were also predicted and verified experimentally using flow cytometry based membrane potential measurements. Pareto analysis of NADH and ATP maintenance showed the decoupling of electron transfer and ATP synthesis in StrpR. Redox homeostasis and NAD^+^ cycling through rewiring metabolic flux was implicated in re-sensitizing antibiotic resistant *C*. *violaceum*. These approaches can be used to probe metabolic vulnerabilities of resistant pathogens. On the verge of a post-antibiotic era, we foresee a critical need for systems level understanding of pathogens and host interaction to extend shelf life of antibiotics and strategize novel therapies.

## Introduction

*Chromobacterium violaceum* is abundantly present in the soil and water as microbiota in tropical and subtropical regions around the world. For many years, it was mainly known as a producer of violacein and as a reporter for the discovery of quorum sensing molecules. In recent years, *C*. *violaceum*, primarily a neglected zoonotic pathogen, has emerged as an important model of an environmental opportunistic human pathogen, with a current low incidence of about 150 cases worldwide [1]. The high mortality rate (ca. 65–80% [2]) of infection with this gram-negative β-proteobacterium, is a result of its unique survival strategies against antibiotics coupled with directed enzyme activities (superoxide dismutase and catalase) to escape host defense [3]. Its high virulence in human infections and a mouse infection model involves the possession of several predicted virulence traits, including two type III secretion systems (T3SSs) [4]. Further, antibiotic resistance is also a critical factor in chronic infection. One of the defining phenotypes of this pathogen is the vivid metallic purple color, attributable to the blue-violet, non-diffusible pigment violacein produced from the essential amino acid tryptophan. Violacein is a virulence factor and also has documented antibiotic properties [5]. The production of violacein is easy to screen and allows one to use this as a model system to study the emergence and control of antibiotic resistance. Understanding complex relationships between genotype and phenotype, using GSMM has become increasingly fundamental to systems biology of pathogens. Availability of the genome sequence of *C*. *violaceum* [6] mandates a functional GSMM, as has been reconstructed for multiple pathogens [7–9]. Such a GSMM would potentially enable both metabolic engineering of violacein and elucidation of pathogenic and resistance mechanisms.

Antibiotic resistance has become a grand challenge for society and a multipronged approach that addresses surveillance, awareness and scientific mechanisms are required to combat it [10]. Our previous study integrating genomics, limited phenomics and metabolomics data [11], unraveled disruption of critical redox homeostasis by specific metabolite supplementation causing death of streptomycin resistant (StrpR) and chloramphenicol resistant (ChlR) populations of *C*. *violaceum*. It also highlighted the utility of a core model of metabolism integrated in the context of systems-level data, to generate hypothesis and predict emergent properties of antibiotic resistance.

In this study, the *C*. *violaceum* genome sequence was used to develop a GSMM through an initial automated draft reconstruction using the Model SEED server. The draft reconstruction was translated into a functional mathematical model after manual curation with legacy data and customized biomass macromolecular composition. The model was validated (prediction accuracy of 89%) using legacy BIOLOG data for respiration. Growth on thirty metabolites were predicted and validated experimentally. Glucose uptake rates (GUR), violacein secretion rates (VSR) and ATP maintenance (ATPM) were used as constraints to customize models to represent antibiotic resistant and susceptible phenotypes. Flux variability analysis (FVA) showed metabolic flux redistribution associated with antibiotic resistance in reactions involving redox factors NADH, TCA cycle, glyoxylate shunt and overflow metabolism. FVA also identified fold changes in proton motive force through ATP synthesis proportional to that observed in PMF using flow cytometer. Pareto front analysis was performed to identify tradeoffs between ATP and NADH maintenance using an *in silico* NADH oxidase reaction (NOX) in the growth of the resistant populations *vis a vis* wild type (WT). This study represents *C*. *violaceum in silico* and correlates it’s metabolic features to antibiotic resistance and predicts related metabolite vulnerabilities. Such approaches could lead to scalable pipelines using OMICS derived constraints-based flux balance models for clinical isolates.

## Results

### Genome scale reconstruction and model statistics

The draft reconstruction was obtained from Model SEED based on genome sequence of ATCC 12472 strain [6] and contained 1303 reactions, 1144 metabolites and 892 genes. Of the 4407 protein coding genes 61.3% were annotated for function, of which 20% were in the draft model. The reconstruction was transformed into a functional model with 1255 reactions and 971 metabolites and 858 genes representing *C*. *violaceum* metabolism. The curation involved systematic literature mining. Data mining through PubMed search engine resulted in 750 research articles related to *C*. *violaceum* (Fig A in S1 File). 472 papers provided evidence for gene protein reaction relationships and helped in model curation.

**Fig 1:**
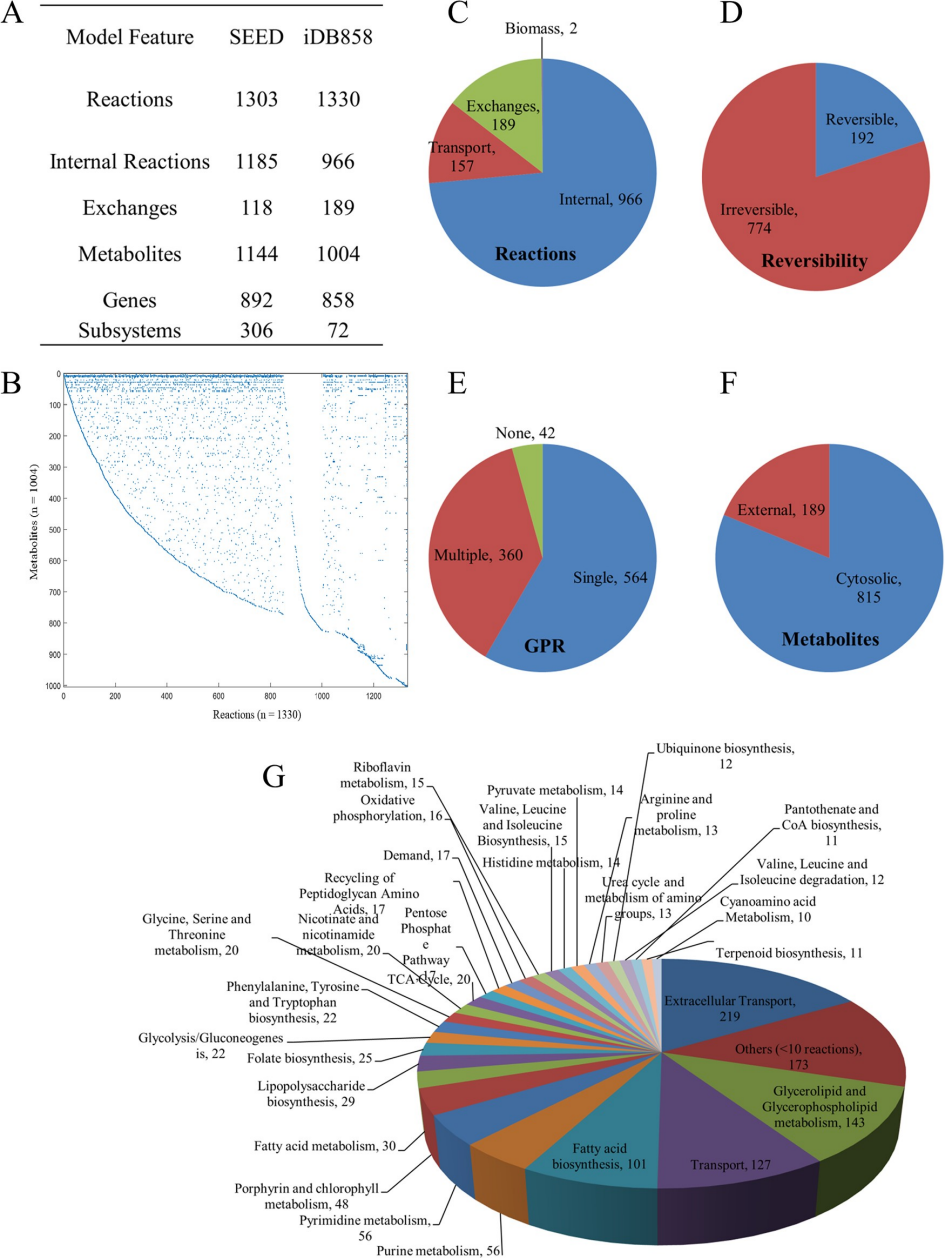
Model statistics and subsystem wise classification for *i*DB858. (A) Table for Model characteristics (B) A dot plot of the stoichiometric matrix for *i*DB858 with all 1330 reactions represented on X-axis and all 1004 metabolites on Y-axis. Each nonzero value is represented by a dot. (C) to (F) Pie charts representing categorization of reactions, reaction reversibility, gene protein associations (GPR) and metabolites, respectively. (G) Subsystems wise classification of the reactions present in the model.

The SEED-derived *in silico C*. *violaceum* was unable to produce twenty six out of 74 biomass precursors [12,13] with glucose as substrate. Sixty-nine reactions (Table E in S1 File) were added to allow biomass formation *in silico* based on experimental evidence for *C*. *violaceum* or phylogenetically related *N*. *meningitidis* (Tables B to D in S1 File). Twenty metabolites were added with “mDB” prefix and 143 reactions with “rDB” prefix. The average confidence score for the model was 1.45. The model statistics for *i*DB858 is presented in Fig 1. The model *i*DB858 successfully predicted the physiology of *C*. *violaceum* as per legacy data (Table 1). A detailed description of the *in silico* representation of metabolic genome features of *C*. *violaceum* have been provided in Material B in S1 File.

**Table 1:** Physiological characteristics successfully predicted by *i*DB858.

Physiological function		*In silico*	Experimental Reference
Lactate utilization		+	Ron Taylor 2009
Acetonitrile utilization		+	Chapatwale 1988
Glycerol utilization			In house
Violacein production		+	Lichstein and Van de Sand 1945
Cyanide production	Glucose	+	Michaels and Corpe, 1965
	Succinate	+	
	Glutamate	++	

### SEED draft model limitations

The SEED reconstruction did not reflect the complete biosynthetic pathways of amino acids and nucleotides present. Violacein biosynthesis (Table F in S1 File) critical and characteristic of *C*. *violaceum* [14] was also missing. The details of missing reactions added to the model are provided in Table A and Material A in S1 File. Thus automated draft reconstructions need detailed manual curation for refined reconstructions that can be translated to models to compute cell phenotype accurately.

### Metabolic capacity validation of *i*DB858 based on BIOLOG GN2 plate phenotype

**Fig 2:**
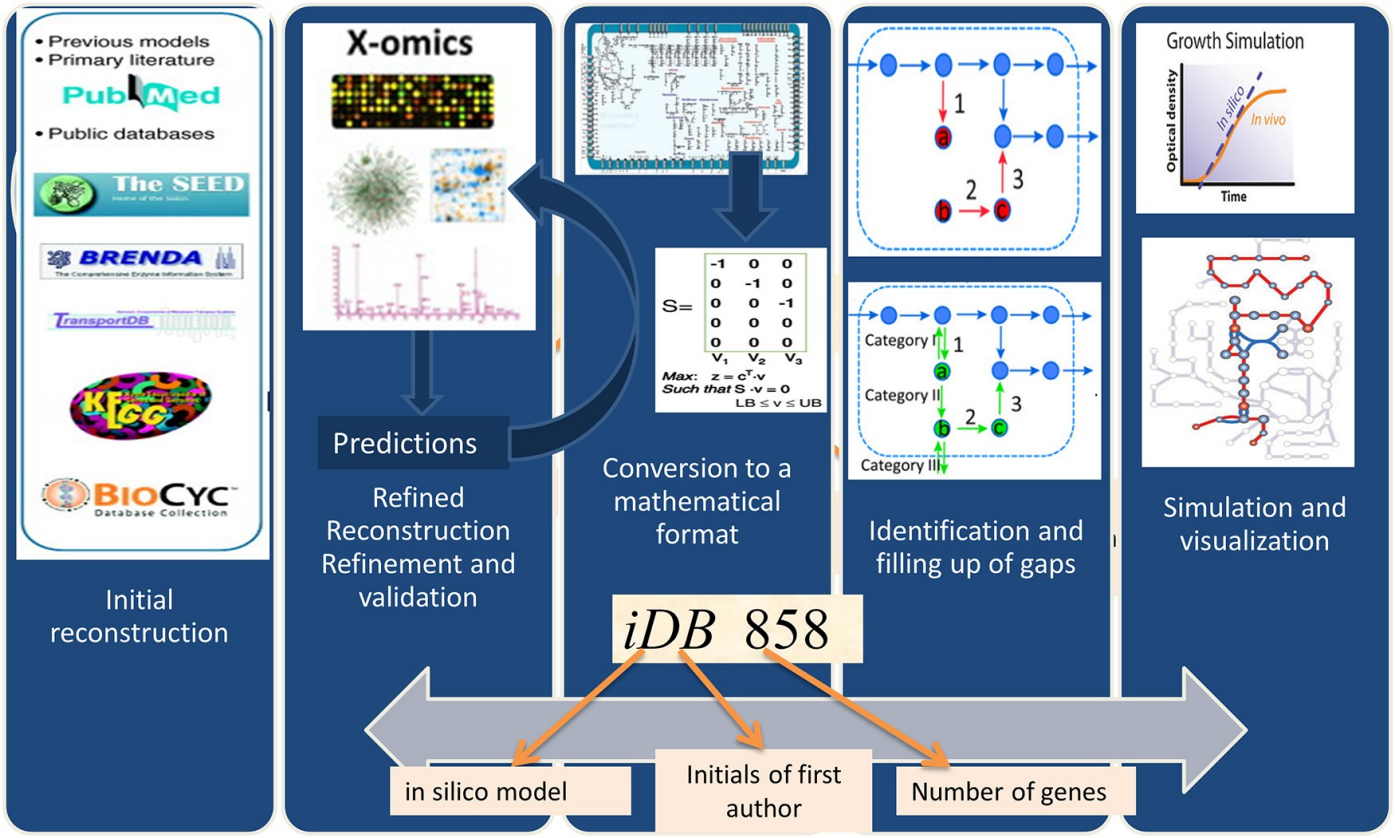
Reconstruction of genome scale metabolic model.

**Fig 3:**
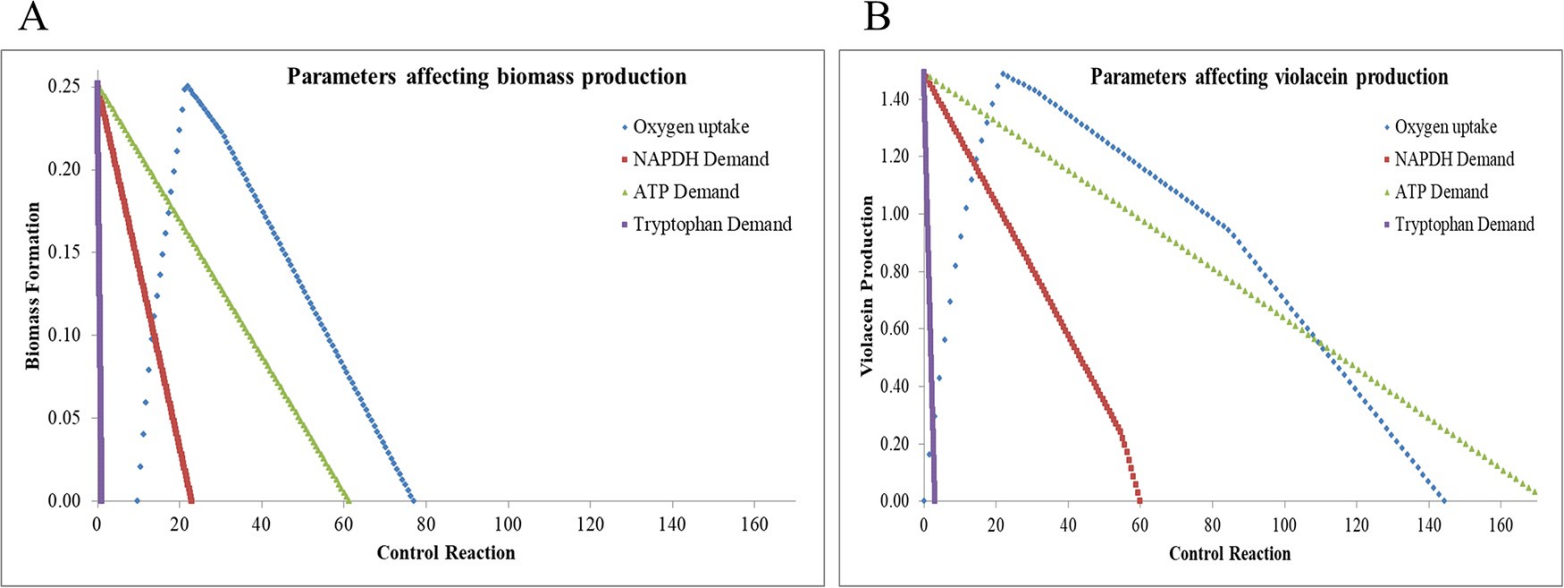
Robustness analysis. To understand metabolite limitation on biomass (A) and violacein (B) formation in *i*DB858 in glucose.

The summarized metabolic reconstruction and modeling process is iterative (Fig 2). The GSMM reconstruction was converted into a model to compute growth/respiration phenotypes on several C/N sources. Predictions were validated using experimental high-throughput phenotypic array data (Biolog^™^) [15–17]. Failure modes were used to refine the model by adding 36 missing reactions. *i*DB858 was able to predict metabolic phenotypes of *C*. *violaceum* with a prediction accuracy of 89% (Table 2 and Table G in S1 File). Sensitivity of violacein biosynthesis to oxygen, NADPH, ATP demands and tryptophan were delineated using robustness analysis (Fig 3) to identify tryptophan and NADPH as bottlenecks using *i*DB858.

**Table 2:** BIOLOG *in silico* prediction accuracy by *i*DB858.

Total Substrates	95	
Not in Model	38	
Present in Model	57	
True Positive	37	
True Negative	12	
False Positive	2^a^	^a^Glycerol and Formate
False Positive	4^b^	^b^D-serine, Uridine, 2-Aminoethanol, Cis-aconitate
False Negative	2^b^	^b^Leucine, Glucose-1-phosphate

^**a**^Experimental evidence exists,

^**b**^Conflicting literature evidence

### Growth prediction accuracy and ATP maintenance

*i*DB858 predicted growth on 24 out of 27 exogenous metabolites tested (88.9% accuracy; Table 3 and Table H in S1 File). The inability to utilize citrate, oxalic acid and glyceraldehyde-3-phosphate was ascertained.

**Table 3:** Exogenous metabolites *in silico* prediction accuracy by *i*DB858.

Total Substrates	30
Not in Model	1
Present in Model	29
True Positive	22
True Negative	2
False Negative	5^a^

^a^Mannitol, Sorbitol, Tryptophan, Valine and Glutamine

The energy and maintenance requirements for instantaneous steady state vary when accounting for violacein yields in addition to biomass yields. The ATP maintenance requirements for wild type *C*. *violaceum* (WT) growing on glucose with violacein secretion decreased the maintenance costs (45%) for biomass synthesis from 12.59 to 6.96 mmol ATP per g of biomass. As a consequence if the violacein demand decreases, the ATP maintenance costs would increase. For wild type and resistant strains (ChlR and StrpR) and experimental conditions described here (Table 4), the non-growth associated maintenance requirement was determined to be 6.96, 10.67 and 6.77 mmol of ATP per g of biomass respectively. This indicates lower violacein yields (as observed) for ChlR. Although StrpR had similar ATP maintenance costs, increased experimental yields of violacein indicate reprogramming of metabolism to compensate. Growth of WT *i*DB858 was under predicted by 19% and over predicted for ChlR and StrpR at fixed optimal oxygen uptake rates. Thus modulation of violacein yields and energy maintenance costs may be a metabolic signature of the action of these antibiotics. Similarly the ATP maintenance requirements for WT on pyruvate, succinate and D-malate were calculated as 3.62, 4.94 and 2.6 ATP per g of biomass respectively.

**Table 4:** Experimental constraints used to define the three different population of *C*. *violaceum*.

Model	Glucose uptake rate^a^	Violacein secretion rate^a^	Molar growth yield^a^	ATPM	Biomass (*in-silico*)	Biomass (*in-vitro*)^a^	Oxygen uptake rate
WT	9.99	1.49	0.0312	6.96	0.25	0.31	21.58
ChlR	10.53	0.673	0.0314	10.67	0.68^b^	0.33	9.91
StrpR	12.78	0.702	0.0504	6.77	0.92^b^	0.64	17.03

^a^Experimental values.

^b^Constrain oxygen to lower the biomass predicted to match experimental biomass

### Redox coupled metabolic flux redistribution a function of antibiotic perturbation in *C*. *violaceum* metabolic network

**Fig 4:**
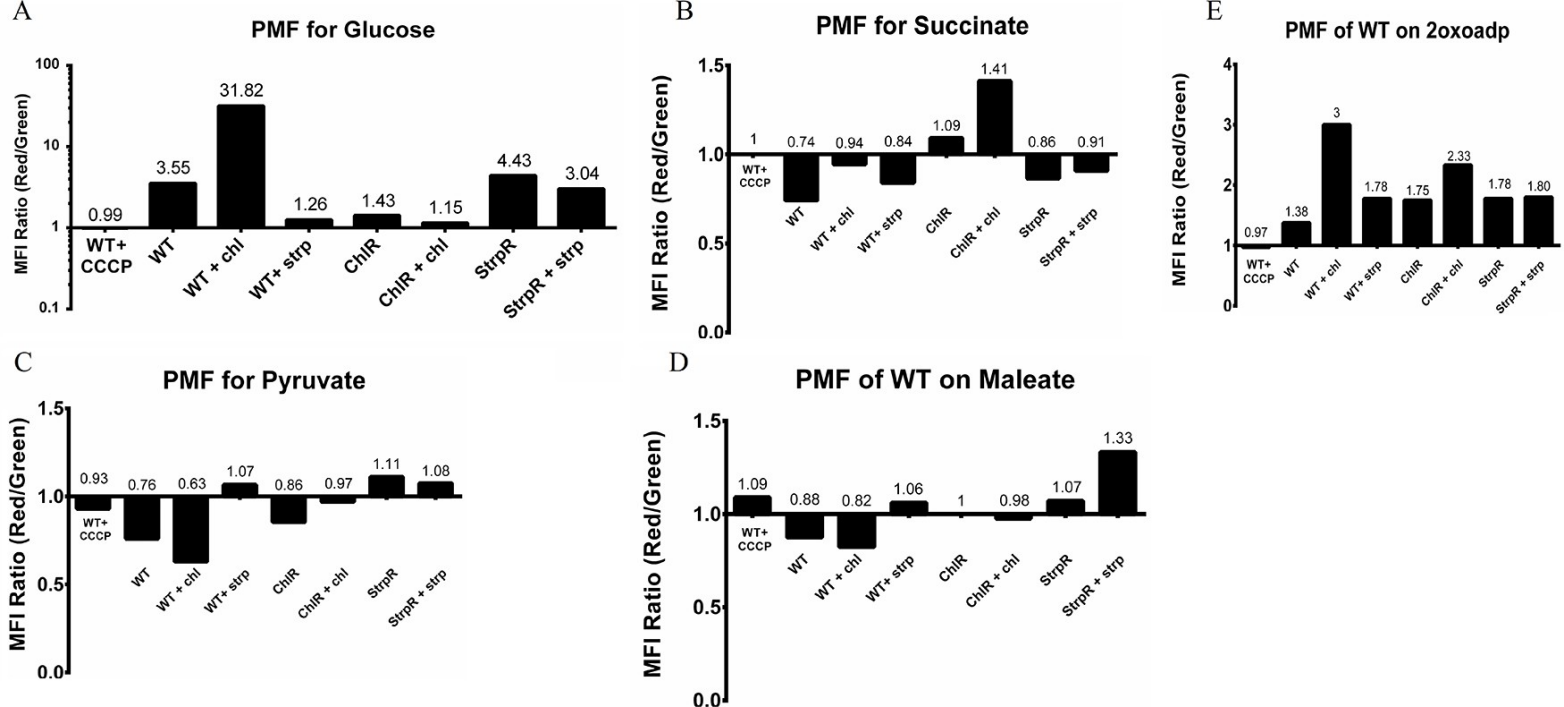
Proton motive force (PMF) analysis using flow cytometry based membrane potential measurements for different metabolites including glucose, succinate, pyruvate, maleate and 2oxoadipate. More than three replicates were used with a standard deviation between 0.075 (glucose) to 0.35 (2 oxoadipate).

Flux variability analysis (FVA) was used to show perturbed central metabolism and redox balance in the presence of antibiotics and reprogrammed metabolism as compensatory mechanisms in resistant populations. To understand the effect of antibiotics on *C*. *violaceum* cellular metabolism the changes in feasible metabolic flux distributions in the presence of chloramphenicol (WT+chl) and streptomycin (WT+strep) were delineated using FVA and analysed (Tables 5 and 6). Altered metabolism in WT in presence of chloramphenicol include overflow metabolism via secretion of acetate and formate. The rewiring of pyruvate formate lyase (PFL) to function unidirectionally leads to formate accumulation. Fumarate reductase (FRD7) carries a very negligible flux. FRD7 represents TCA as well as oxidative phosphorylation and is involved in relaying electrons towards cytochrome oxidase that eventually creates a PMF/ electrochemical membrane gradient for ATP synthesis. The failure of FRD7 to remain a control node in the presence of chloramphenicol indicates the continued use of O_2_ as terminal electron acceptor. The corresponding Electron Transport Chain (ETC) complex, represented by cytochrome oxidase bo3 carries a 12 fold lower flux compared to WT. Experimentally measured PMF using flow cytometry based membrane potential measurements is 10 fold higher measured (Fig 4) in the presence of chloramphenicol through potential disruption of the lipid bilayer and increased proton pumping through formate and acetate [18].

**Table 5:** Flux variability analysis (FVA) to show the effect of chloramphenicol on WT.

Subsystem	Reaction ID	Reaction Formula	WT	WT+chl
Glycolysis	PYK	adp_c + pep_c -> atp_c + pyr_c	0.0005	0.76
TCA Cycle	FRD7	succ_c + q8_c < = > fum_c + q8h2_c	5.13	-0.00039 to 0.00019
Oxidative phosphorylation	cytochrome oxidase bo3 ubiquinol-8	2.5 h_c + 0.5 o2_c + q8h2_c -> h2o_c + 2.5 h_e + q8_c	31.22	2.61
Pyruvate metabolism	PFL	accoa_c + for_c < = > coa_c + pyr_c	-10.84 to 0.23	-0.45
	PTAr	h_c + accoa_c + pi_c -> actp_c + coa_c	0.0001	1.53
	ACKr	actp_c + adp_c -> h_c + atp_c + ac_c	0.0001	1.53
	EX_for	for_e < = >	0.0001	0.45
	EX_ac	ac_e < = >	0.0001	1.53

For reaction details and color code for the FVA category refer to S1 Table.

**Table 6:** Flux variability analysis (FVA) to show the effect of streptomycin on WT.

Subsystem	Reaction ID	Reaction Formula	WT	WT+strep
TCA Cycle	AKGDH	coa_c + nad_c + akg_c -> co2_c + nadh_c + succoa_c	0.05	1.10
	FUM	mal__L_c < = > fum_c + h2o_c	-5.31	-3.14
	MDH	nad_c + mal__L_c < = > h_c + nadh_c + oaa_c	5.72	2.04
Oxidative phosphorylation	cytochrome oxidase bo3 ubiquinol-8	2.5 h_c + 0.5 o2_c + q8h2_c -> h2o_c + 2.5 h_e + q8_c	31.22	10.03
Pyruvate metabolism	PFL	accoa_c + for_c < = > coa_c + pyr_c	-10.84 to 0.23	-1.88 to 1.1
	PPS	atp_c + h2o_c + pyr_c -> h_c + pi_c + pep_c + amp_c	0.0002	0.15
Purine metabolism	ATP carbamate phosphotransferase	atp_c + co2_c + nh4_c < = > h_c + adp_c + cbp_c	0.17	1.10
Folate biosynthesis	MTHFD	nadp_c + mlthf_c < = > nadph_c + methf_c	0.41	1.10
	FTHFD	h2o_c + 10fthf_c -> h_c + for_c + thf_c	0.22	1.10
Glutamate metabolism	ASPTA	asp__L_c + akg_c < = > oaa_c + glu__L_c	-0.63	-1.10
Glycine, Serine and Threonine metabolism	PSERT	akg_c + pser__L_c < = > glu__L_c + 3php_c	-3.54	-1.10
	GHMT	gly_c + h2o_c + mlthf_c < = > ser__L_c + thf_c	-0.46	-1.10
Arginine and proline metabolism	PRO1x	h_c + nadh_c + 1pyr5c_c -> nad_c + pro__L_c	0.35	0.002
	SOTA	akg_c + sucorn_c < = > sucgsa_c + glu__L_c	4e-5	1.10
	SGSAD	h2o_c + nad_c + sucgsa_c -> 2 h_c + nadh_c + sucglu_c	4e-5	1.10
	SGDS	h2o_c + sucglu_c < = > succ_c + glu__L_c	4e-5	1.10
	AST	arg__L_c + succoa_c -> h_c + coa_c + sucarg_c	4e-5	1.10
Urea cycle and metabolism of amino groups	ARGSL	argsuc_c -> fum_c + arg__L_c	0.07	1.10
	ARGSS_1	atp_c + asp__L_c + citr__L_c -> ppi_c + argsuc_c + amp_c	0.07	1.10
	AGGPR	nadph_c + acg5p_c -> pi_c + nadp_c + acg5sa_c	0.08	1.10
	OCBT	cbp_c + orn_c -> 2 h_c + pi_c + citr__L_c	0.07	1.10
	ORNTAC	glu__L_c + acorn_c < = > orn_c + acglu_c	0.08	1.10
	ACGK	h_c + atp_c + acglu_c -> adp_c + acg5p_c	0.08	1.10
	ACOTA	glu__L_c + acg5sa_c -> akg_c + acorn_c	0.08	1.10
Cyanoamino Metabolism	glycine:acceptor oxidoreductase	gly_c + 2 nadph_c -> co2_c + 2 nadp_c + hcn_c	0.28	1.10
	cyn_rxn6	hcn_c -> acybut_c	0.28	1.10
	NH4+ Exchange	nh4_e < = >	-6.28	1.09

For reaction details and color code for the FVA category refer to S1 Table.

In the presence of streptomycin however, higher ammonia and cyanide are produced and siphoned off to make more glutamate and glycine as seen experimentally and varied folate metabolism (through increased p-aminobenzoic acid) [11]. Lysine, methionine, histidine correlated to the altered function (via mutation) of the PLP utilizing PabC. Glutamate efflux (experimental data and Succinylornithine transaminase, SOTA reaction directionality in Table 6) have been implicated previously in streptomycin induced decreased cell viability [19]. These results are validated by the metabolite profiling data from our previous study [11]. The increased flux towards tetrahydrofolate synthesis with co-synthesis of formate (Formyltetrahydrofolate amidohydrolase, FTHFD in Table 6) is potentially rechanneled through the folate pathway instead of formate secretion. Around 5% (68) of the total reactions including 11 redox coupled reactions spanning 16 different subsystems showed differential flux distribution for the antibiotic presence (Fig F in S1 File). These 11 redox coupled reactions (involving NADH/NADPH) show that there is increased accumulation of NADH in the presence of streptomycin as compared to chloramphenicol that was confirmed by experimental quantitation of NAD/NADH levels [11]. This could potentially lead to pseudo-hypoxia even in the presence of normal oxygen levels. These changes observed *in silico* as well as *in vitro*, confirms modulation of flux involving NADH. FRD7 is reduced 2.5 fold. ATP generation is almost three fold lower through the ETC-PMF route similar to that of WT. The PMF is a third of that observed in the wild type (Fig 4) and correlates well to the 3 fold decrease in unique flux held by the cytochrome oxidase bo3 reaction.

### Compensatory metabolic reprogramming as a survival strategy in resistant population

The metabolic reprogramming due to antibiotic selection pressures was analysed by FVA using customized models to represent WT, ChlR and StrpR (Tables 7 and 8). Differences in unique forced fluxes/rates between resistant and susceptible populations in central metabolic pathways indicate compensatory metabolic reprogramming. Both the resistant populations resort to overflow metabolism towards acetate. Isocitrate lyase (ICL), the glyoxylate shunt enzyme implicated in pathogenesis and persistence in *Salmonella* and resistance in *Mycobacterium* [20] shunts isocitrate by bypassing part of the TCA cycle. The glyoxylate shunt functions in case of StrpR but TCA continues through oxidative branch in case of ChlR. In case of StrpR higher flux through pyrimidine metabolism is represented by cytidylate kinase (CYTK1 in Table 8) that correlated well with previously published liquid chromatography—mass spectrometry (LCMS) data showing higher average relative flux of cytosine and adenosine in StrpR [11]. The flux through cytochrome oxidase (oxidative phosphorylation) is reduced by 50%. This potentially reduced the membrane potential by half (Fig 4) as captured by flow cytometry analysis. Lowering iron homeostasis has been recently implicated as one mechanism that plays a critical role in antibiotic mediated cell death and evolution of *de novo* antibiotic resistance [21,22]. The iron related oxygen oxidoreductase reprogrammed to increase Fe^+2^ indicates lower probability of Fenton reaction and DNA mutations. The lowered oxygen uptake rate and the increased flux towards NADH, potentially represent a pseudohypoxic state. Lowered ratios of NAD+/NADH increase pyruvate dehydrogenase (PDH) phosphorylation and modulate pyruvate and acetate levels. In the presence of chloramphenicol, the ChlR population growing on glucose maintained the NAD/NADH ratio at 0.28 while in the presence of streptomycin, the StrpR population maintained the NAD/NADH ratio at 2.47. Potentially, the PDH must be completely dephosphorylated in the ChlR (supporting acetate and formate overflow) as compared to StrpR (only acetate overflow) to evoke such a response.

**Table 7:** Flux variability analysis (FVA) to show compensation in case of ChlR.

Subsystem	Reaction ID	Reaction Formula	WT	ChlR
Glycolysis	PYK	adp_c + pep_c -> atp_c + pyr_c	0.001	1.44
TCA	SUCOAS	atp_c + coa_c + succ_c -> adp_c + pi_c + succoa_c	4e-5	0.07
	MDH	nad_c + mal__L_c < = > h_c + nadh_c + oaa_c	5.72	0.22
	ICDHyrb	nadp_c + icit_c < = > h_c + mDB_oxasucc_c + nadph_c	0.00005	0.0038
Oxidative phosphorylation	cytochrome oxidase bo3	2.5 h_c + 0.5 o2_c + q8h2_c -> h2o_c + 2.5 h_e + q8_c	31.22	14.42
Pyruvate metabolism	PTAr	h_c + accoa_c + pi_c -> actp_c + coa_c	0.0001	11.78
	ACKr	actp_c + adp_c -> h_c + atp_c + ac_c	0.0001	11.78
	PFL	accoa_c + for_c < = > coa_c + pyr_c	-10.84 to 0.23	-9.58
	PPC	co2_c + h2o_c + pep_c -> 2 h_c + pi_c + oaa_c	0.0001	0.60
Glyoxylate and dicarboxylate metabolism	ICL	icit_c < = > succ_c + glx_c	5.09	-0.004
Purine metabolism	ADK2	h_c + amp_c + pppi_c -> ppi_c + adp_c	0.0007	0.001
Pyrimidine metabolism	CYTK1	atp_c + cmp_c -> adp_c + cdp_c	0.06	0.08
Porphyrin and chlorophyll metabolism	FeII oxygen oxidoreductase	4 h_c + o2_c + 4 fe2_c < = > 2 h2o_c + 4 fe3_c	0.00005	-0.0007
Extracellular Transport	EX_ac_e	ac_e < = >	0.0001	11.82
	EX_for_e	for_e < = >	0.0001	9.80

For reaction details and color code for the FVA category refer to S1 Table.

**Table 8:** Flux variability analysis (FVA) to show compensation in case of StrpR.

Subsystem	Reaction ID	Reaction Formula	WT	StrpR
Glycolysis/Gluconeogenesis	PYK	adp_c + pep_c -> atp_c + pyr_c	0.001	1.61
TCA Cycle	AKGDH	coa_c + nad_c + akg_c -> co2_c + nadh_c + succoa_c	0.05	0.14
	MDH	nad_c + mal__L_c < = > h_c + nadh_c + oaa_c	5.72	1.58
Oxidative phosphorylation	cytochrome oxidase bo3 ubiquinol-8	2.5 h_c + 0.5 o2_c + q8h2_c -> h2o_c + 2.5 h_e + q8_c	31.22	28.42
Pyruvate metabolism	PTAr	h_c + accoa_c + pi_c -> actp_c + coa_c	0.0001	11.76
	ACKr	actp_c + adp_c -> h_c + atp_c + ac_c	0.0001	11.76
	ACALD	acald_c + coa_c + nad_c < = > h_c + accoa_c + nadh_c	0.0007	0.002
	PFL	accoa_c + for_c < = > coa_c + pyr_c	-10.84 to 0.23	-14.53 to 0.59
	MALS	accoa_c + h2o_c + glx_c -> h_c + coa_c + mal__L_c	5.10	0.51
	ME2	nadp_c + mal__L_c -> co2_c + nadph_c + pyr_c	4.69	0.00004
	PPC	co2_c + h2o_c + pep_c -> 2 h_c + pi_c + oaa_c	0.0001	0.54
Glyoxylate and dicarboxylate metabolism	ICL	icit_c < = > succ_c + glx_c	5.09	0.50
Pyrimidine metabolism	CYTK1	atp_c + cmp_c -> adp_c + cdp_c	0.06	0.16
Porphyrin and chlorophyll metabolism	FeII oxygen oxidoreductase	4 h_c + o2_c + 4 fe2_c < = > 2 h2o_c + 4 fe3_c	0.00005	-0.001
Extracellular Transport	EX_ac_e	ac_e < = >	0.0001	11.85

For reaction details and color code for the FVA category refer to S1 Table.

The NAD/NADH ratios in the wild type were around 0.25 for glucose and pyruvate, while it was 0.73 for succinate and oxoadipate. The ChlR strain shows major 7 fold reduction indicating high levels of NAD recycling provided by pyruvate. Concurrently, PMF/membrane potential is higher for the resistant populations in pyruvate as compared to the wild type. This suggests potential incapability to maintain ATP homeostasis under these conditions and an eventually complete decoupling of electron transfer and ATP synthesis. Similar results were observed for succinate, maleate and 2-oxoadipate.

### Decoupling NADH oxidation from respiratory energy generation using in silico water forming NADH oxidase (NOX)

NADH oxidase (NOX) allows dissecting the role of redox imbalance and ATP synthesis deficiency in ETC function [23]. The flux through NOX (at constant GUR) indicates the need to recycle excess 13.2 and 10.31 mmol NADH per gDW of biomass for redox balance in ChlR and StrpR respectively (Table 9). This underscores the importance of NAD+ recycling to maintain growth in both resistant populations. Since converting NADH to NAD+, NOX also consumes protons and oxygen, oxygen consumption (OUR) in ChlR and StrpR strains increased by 170% and 86% respectively. In the absence of NOX, the reduced apparent OUR indicate pseudo-hypoxia as previously reported [24]. Although the ATPM for StrpR is similar to that of WT (6.77 compared to 6.96), a significant flux (10.31) through NOX is essential to attain experimental growth yields. This suggests decoupled redox homeostasis and PMF in StrpR.

**Table 9:** Constraints used for NADH oxidase (NOX) simulations for ChlR and StrpR.

Model	Glucose uptake rate	Violacein secretion rate	ATPM	Biomass		Oxygen uptake rate	NOX
				(*in-silico*)	(*in-vitro*)		
ChlR	10.53	0.673	10.67	0.68	0.33	9.91	0
				0.68	0.33	26.53	13.2
StrpR	12.78	0.702	6.77	0.92	0.64	17.03	0
				0.92	0.64	31.7	10.31

FVA of the three populations (WT, ChlR and StrpR) in presence of NOX provided an insight into NAD+ recycling associated metabolic reprogramming in the resistant populations. Based on the feasible flux distributions, the resistant populations behaved very similar to the wild type (Table 10). Metabolic reprogramming via NAD recycling (Table 10, yellow) were identified along with overflow metabolism. Introduction of NOX alone in StrpR supported WT-like phenotypes but for ChlR (Table 10, red) varied ATPM maintenance was also critical. This confirms decoupled redox electron transfer and ATP synthesis in StrpR.

**Table 10:** Flux variability analysis (FVA) category changes post NADH oxidase (NOX) addition to ChlR and StrpR models of *C*. *violaceum*.

Subsystem	Reaction ID	WT	ChlR^d^	StrpR^d^	ChlR^e^	StrpR^e^	ChlRN ox^d^	StrpR Nox^d^	ChlR Nox^e^	StrpR Nox^e^
TCA Cycle	MDH^a^	2	1	1	2	1	2	2	2	2
	CS^b^	1	7d	1	7d	1	1	1	1	1
	SUCOAS^b^	7d	1	7d	1	7d	7d	7d	7d	7d
	FRD7^b^	2	8	2	8	2	2	2	2	2
	AKGDH^b^	3	7d	3	7d	3	3	3	3	3
Pyruvate metabolism	ME2^a^	3	7d	7d	7d	7d	3	3	3	3
	PPC^a^	7d	1	1	2	1	7d	7d	7d	7d
	OAADC^a^	3	7d	7d	7d	7d	3	3	3	3
	PFL^b^	8	5	8	5	8	8	8	8	8
	MALS^b^	1	7d	1	7d	1	1	1	1	1
Glyoxylate & dicarboxylate metabolism	ICL^b^	1	4	1	4	1	1	1	1	1
Glutathione metabolism	AMPTASECG^c^	7b	5	4	7b	4	4	4	7b	4
	glutathione hydralase^c^	7b	5	4	7b	4	4	4	7b	4
Extracellular Transport	Ex_for_e^b^	7d	1	7d	2	7d	7d	7d	7d	7d

For FVA category color code refer to S1 Table.

^a^Reactions common to both resistant population

^**b**^Reactions unique to ChlR

^**c**^Reactions unique to ChlR when WT ATPM was used

^d^FVA using their respective ATPM values

^e^FVA using the WT ATPM value

### Gene essentiality and synthetic lethality

**Fig 5:**
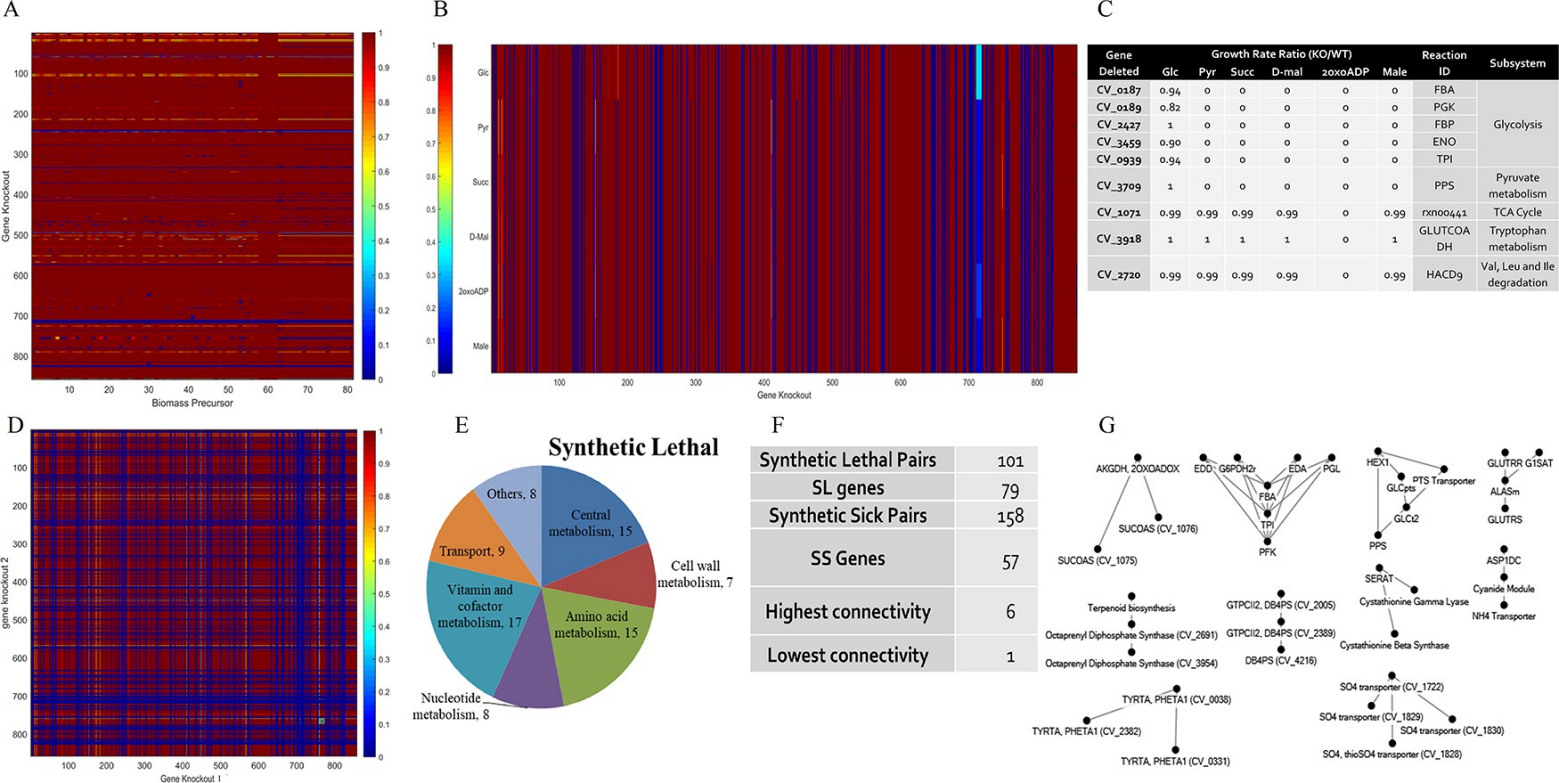
Gene deletion analysis. (A) Heat map for single gene deletion (SGD) analysis for biomass precursors. (B) Heat map and table (C) for SGD analysis for 5 candidate metabolites along with glucose. (D) Heat map for double gene deletion (DGD) analysis on glucose under aerobic condition. (E and F) Unique genes involved in synthetic lethal pair interaction during DGD analysis. (G) Synthetic lethal pair interactions for two or more connectivity, highest connectivity observed in case of upper glycolysis and Entner Duordoff pathway.

Gene essentiality analysis for growth and biomass precursors (Fig 5A) identified 191 virulent genes (Table 11) minimally required for survival in glucose medium aerobically. 644 genes were predicted as avirulent and 23 attenuated genes resulting in 36% to 98% reduction in growth. Conditional dependent essentiality was assessed on C-source metabolites pyruvate, succinate, maleate, D-malate and 2-oxoadipate (Fig 5B and 5C), candidates for re-sensitization of ChlR and StrpR to antibiotics. 191 genes belonging to different subsystems were predicted essential for glucose and other substrates (Fig E in S1 File). Nine essential genes were substrate independent (Fig 5C). These included five genes from glycolysis and anaplerosis (for pyruvate). 3 additional genes essential for growth on 2-oxoadipate were a part of TCA, tryptophan metabolism and valine, leucine and isoleucine degradation subsystem.

**Table 11:** Single gene deletion analysis of *i*DB858 on glucose under aerobic condition.

Category	GR Ratio	Genes
Attenuated	0.36 to 0.98	23
Virulent genes	0	191
Avirulent genes	0.99 to 1	644

Double gene deletion (DGD) analysis (Fig 5D to 5G) led to identification of 186 genes in 518 combinations resulting in synthetic lethal (SL) and sick (SS) interactions. Synthetic lethal and sick interactions predicted that 129 genes involved in synthetic lethal pairs were related to porphyrin metabolism, phenylalanine, tyrosine and tryptophan biosynthesis and purine metabolism. CV_0939, *tpiA* had the highest connectivity. CV_1071 involved in utilization of 2oxoadipate (resensitisation candidate) utilization was synthetically lethal with two genes from the TCA cycle (CV_1075 or CV_1076). This might explain the criticality of TCA (central metabolism) in resensitising the resistant population to 2 oxoadipate. The synthetic lethal pairs can be utilized for predicting drug targets that would otherwise be extremely challenging (possible pairs based on 858 genes—7,36,164) to test experimentally. All the simulation results and *i*DB858 model have been provided in supporting information.

## Discussion

Antibiotic lethality may be initiated by altered cellular redox state. The redox stress potentially rewires central metabolism, cellular respiration, and iron resulting in cellular damage [25]. Our work exemplifies how systems-level analyses can help dissect the complexity involved in oxidative phosphorylation and electron transfer in responses to drugs.

A whole genome-scale metabolic model of *Chromobacterium violaceum*, *i*DB858 was generated that predicted response to antibiotics and metabolic reprogramming in resistant populations. FVA identified rewired central and redox metabolism in the presence of antibiotics and resistant populations all represented by *in silico* constraints. The relative contributions of redox imbalance and ATP insufficiency contributing to antibiotic action were delineated. *i*DB858 showed all redox coupled reactions carried uniquely determined flux confirming the critical role of redox homeostasis in maintaining viability and cellular function in *C*. *violaceum* as seen experimentally [25–29]. This also correlated well with the differential NADH/NAD ratios obtained for the resistant and susceptible populations [11]. The metabolic rewiring in *i*DB858 as a response to chloramphenicol or streptomycin resulted in overflow metabolism. Compensations in resistant populations included overflow metabolism to acetate and formate in ChlR but only acetate in StrpR. StrpR bypassed the oxidative branch of TCA and redirected metabolism to the glyoxylate shunt (ICL, MALS) seen in *Salmonella* persisters.

Addition of NOX (essentially a sink for NADH) to *i*DB858 also captured for the first time the metabolic flux distributions related to NAD+ recycling. This is key for survival of ChlR and StrpR *in silico*. The Pareto optimality analysis of ATP and NADH maintenance in *i*DB858 identified decoupled electron transfer and ATP synthesis in case of StrpR but not in ChlR. This is first of a kind study that shows such decoupling in ETC function in aminoglycoside antibiotic resistant populations.

Although we were able to understand re-sensitization of resistant population in presence of 2-oxoadipate by synthetic lethal pair analysis, further experimental validation is critical to identify more such candidates for inducing death in *C*. *violaceum*.

Quite recently constraints-based flux balance analysis of metabolic models is gaining momentum to understand “emergent phenomena” of antibiotic resistance [11,30–32]. In our knowledge this is the first study that uses genome-scale models and specific redox based objectives to identify metabolic reprogramming and redox homeostasis as compensations to antibiotic selections pressures. Integrating growth, metabolite and minimum inhibitory concentration (MIC) profiling along with constraints-based models could potentially be clinically translated to human pathogens such as ESKAPE (*Enterococcus faecium*, *Staphylococcus aureus*, *Klebsiella pneumoniae*, *Acinetobacter baumannii*, *Pseudomonas aeruginosa*, *and Enterobacter* species). Such approaches are scalable and show promise to identify candidate substrates for re-sensitization and understand complex emergent phenomena like antibiotic resistance.

## Materials and methods

### Bacterial strains and growth conditions

*Chromobacterium violaceum* strain ATCC 12472,wild type, (*C*. *violaceum* or WT) was obtained from the American Type Culture Collection Center (ATCC), USA and routinely cultured on Luria- Broth (LB, Hi-Media-M575) at 30 °C with continuous aeration in a shaker incubator set at 180 revolutions per minute (rpm). Antibiotic resistant strains ChlR and StrpR of *C*. *violaceum* were evolved in our previous study [11] under controlled laboratory environments using the two antibiotics, chloramphenicol (chl) and streptomycin (strep) respectively, at sub-lethal concentrations (10 μg/mL) on Luria Bertani agar (LBA) plates. These strains were cultured in LB with antibiotic (10 μg/mL) at 30 °C with continuous aeration in a shaker incubator set at 180 rpm.

### Membrane potential measurements

BacLight Bacterial Membrane Potential Kit (B34950, Invitrogen) was used to measure changes in PMF induced by antibiotic for all three population of *C*. *violaceum* according to the manufacturer’s instructions. Briefly, stationary cells cultured in presence of different metabolites were diluted to 10^6^ CFU/mL and stained with 10 μL of 3 mM DiOC2 (3), incubated for 30 min. Samples were analyzed using a BD LSR Fortessa SORP cell analyzer flow cytometer (Becton Dickinson, San Jose, CA) with optimized settings. The membrane potential was normalized as the intensity ratio of red fluorescence (a membrane potential- dependent signal) and green fluorescence (a membrane potential-independent signal). The measurements were made for Glucose, Pyruvate, 2oxoADP, Maleate and Succinate. Seven replicates were used for glucose with a standard deviation of 0.075 whereas three replicates were used for the other substrates with standard deviations of 0.14, 0.35, 0.095 and 0.145 respectively. Relative PMF was determined in test samples compared to positive control samples (with glucose) and negative control samples (+CCCP).

### Genome annotation

The complete genome sequence and annotation of *C*. *violaceum* ATCC 12472, GenBank accession number AE016825.1 [6], available online at National Center for Biotechnology Information (http://www.ncbi.nlm.nih.gov) was imported into the RAST server (http://rast.nmpdr.org/) for gene calling and annotation with subsequent manual inspection and curation. This information was used in the metabolic network reconstruction and validation processes.

### Reconstruction of *C*. *violaceum* metabolic network

Fig 2 provides an overview of the genome-scale metabolic reconstruction pipeline of *C*. *violaceum*. The protocol for reconstruction of genome-scale metabolic models (GSMM) [33] was followed and the model name, *i*DB858, was based on existing convention for naming GSMMs [34], 858 representing the number of genes. Thermodynamically infeasible cycles (e.g. cycles resulting in free ATP production) were identified and removed and all reactions checked for mass- and charge-balance. The resulting model was then tested by comparing model predictions to available BIOLOG data [15–17]. Reactions manually identified, were added when sufficient evidence was available from experimental data, NCBI, KEGG, MetaCyc, BioCyc, BRENDA and SEED databases.

### Initial draft reconstruction

An initial draft genome-scale reconstruction of *C*. *violaceum* was built using the RAST server and Model SEED Server (http://www.theseed.org/models/) [35,36]. The objective function for biomass in the SEED reconstruction was modified to reflect the actual macromolecular composition. After modifying the nutrient and biomass composition of the model to accurately capture the boundary conditions that define the overall phenotype, the internal network was curated.

### Manual curation for biomass prediction

The initial draft reconstruction downloaded from Model SEED, was not able to generate biomass using minimal media or a richer chemically defined media. 27 precursors of biomass not forming *in silico* were identified through manual curation. The manually added reactions begin with “rDB” prefix in the model.

### Translation to BiGG database format and consistency check

The SEED reactions and metabolites were matched with KEGG reaction IDs represented in KEGG database (http://www.kegg.jp/) or to the IDs available on BiGG database (http://bigg.ucsd.edu/) to maintain acceptable and clear standards of constraint-based models [37]. Gene annotations were converted from peg IDs to the respective CV gene IDs. Various consistency checks were also performed such as for directionality, occurrence of blocked genes, gaps, orphan metabolites as well as mass and charge balance.

### Biomass composition

Biomass biosynthesis was set as a linear combination of the macromolecules protein, DNA, RNA, lipid, peptidoglycan and LPS, considered to account for the overall biomass composition. A detailed calculation of the biomass composition and its assembly using legacy data is mentioned in Tables B and C and complete breakup is available in Table D in S1 File.

### Flux balance analysis (FBA)

Implementation of the GSMM for *C*. *violaceum* and constraints-based analysis was done using Constraints Based Reconstruction and Analysis (COBRA) Toolbox 2.0.2 [38] with MATLAB v 7.11, (R2010b) and TOMLAB/CPLEX v7.7 optimizer. MATLAB codes for all referenced COBRA functions are available at the COBRA’s website (https://opencobra.github.io/). The function optimizeCbModel(), in COBRA toolbox was used to simulate for growth (maximize biomass objective function) and violacein production as reported earlier [11] using flux balance analysis (FBA).

### Validation of the metabolic model

Validation of *i*DB858 was performed in part by simulating for growth and respiration on metabolites that are potential carbon and nitrogen sources. The predictions were validated using legacy (BIOLOG) substrate utilization data. Predictions of the model for simultaneous growth and violacein production on 30 substrates were validated experimentally.

### Metabolic model of WT, ChlR and StrpR populations

A set of constraints that define the antibiotic susceptible WT and the evolved populations (ChlR and StrpR) were used to customize the models to represent antibiotic susceptible and resistant *Chromobacterium*. The constraints used were experimentally measured in our previous study [11] as shown in Table 4 and included Glucose uptake and Violacein secretion rates (GUR, VSR), Growth yields, and ATP maintenance costs associated with molar growth yields of each strain. The specific growth rates were calculated using 1g biomass as basis.

### Robustness analysis

Robustness analysis, using robustnessAnalysis() was used to better understand the sensitivity of growth and violacein phenotype of *C*. *violaceum* to different environmental perturbations such as oxygen uptake, NADPH, tryptophan, ATP demand.

### Flux variability analysis (FVA)

FVA (set up using fluxVariability()) calculates minimum and maximum flux values for each reaction in the model subject to constraints for specific objectives [39]. Differences in antibiotic sensitive and resistant population flux distributions were classified based on flux magnitude and direction defining rigidity or flexibility [11]. Changing directionality of a reversible reaction or modulating magnitude/span of the reaction flux indicated metabolic reprogramming. FVA was performed in the presence and absence of NADH oxidase (NOX).

### NADH oxidase simulations

A reaction representing NADH oxidase (water forming) was added to the model to delineate the role of NADH imbalance and show decoupling of electron transfer via Electron Transport Chain (ETC) and proton pumping for ATP synthesis [23]. This reaction acts essentially as a drain if there is excess NADH in the system and is represented as:

2h_c+2nadh_c+o2_c→2h2o_c+2nad_c (1)

Analysis of Pareto fronts and trade-off between ATP and NADH maintenance reactions was performed by constraining the GUR, VSR and the growth yield to experimental values and maximizing the fluxes through the generic ATPase (ATPM) and NADH oxidase reaction (NOX).

### Gene essentiality and synthetic lethal analysis in *C*. *violaceum*

Gene essentiality or lethality was predicted using the function singleGeneDeletion(), [40]. *In silico* virulent genes are those that are essential for *C*. *violaceum* growth. Non-essential or avirulent genes and attenuated genes lower growth vis a vis wild type on deletion [40]. Epistatic interactions were identified using doubleGeneDeletion() and delineating synthetic lethal (SL) and synthetic sick (SS) gene pairs.

## Supporting information

S1 File Supplemental figures, tables and text. Supporting figures, tables and additional information regarding manual curation, model features and selected simulations of *i*DB858.
(PDF)

S1 Table Supporting large tables and datasets. Supporting information related to the simulations done using the genome scale metabolic model of *C*. *violaceum*, *i*DB858 using COBRA Toolbox as described in material and methods.
(XLSX)

S1 Model *i*DB858. The .mat file for the genome scale model of *C*. *violaceum*, *i*DB858 used on MATLAB platform for all the simulations reported in this manuscript.
(MAT)
