# Supplementary material for: Constraints-based analysis identifies NAD+ recycling through metabolic reprogramming in antibiotic resistant Chromobacterium violaceum
Source: PLoS One. 2019 Jan 4;14(1):e0210008. doi: 10.1371/journal.pone.0210008 (PMC6319732; doi:10.1371/journal.pone.0210008)
Supplement: S1 File — Supporting figures, tables and additional information regarding manual curation, model features and selected simulations of iDB858. (PDF) [file pone.0210008.s001.pdf]

Supporting Information

Constraints-based analysis identifies NAD^+ recycling through metabolic reprogramming in antibiotic resistant *Chromobacterium violaceum*

Deepanwita Banerjee[1] and Anu Raghunathan[1*]

[1] Chemical Engineering Division, CSIR-National Chemical Laboratory, Pune, India 411008

* Corresponding author

E-mail: anu.raghunathan@ncl.res.in

Running Title: Redox homeostasis through rewiring metabolism in resistant *C. violaceum*

List of Supplementary Information:

Supplementary Figures A to G
Supplementary Tables A to P
Material A: SEED Draft model limitation
Material B: *In silico* representation of metabolic genome features of *Chromobacterium violaceum*
Supplementary References

Figure A: Details about initial draft reconstruction and manual curation

a) Details of job submitted to RAST Server

b) The Model SEED server used to build the initial draft reconstruction

c) Literature mining for manual curation

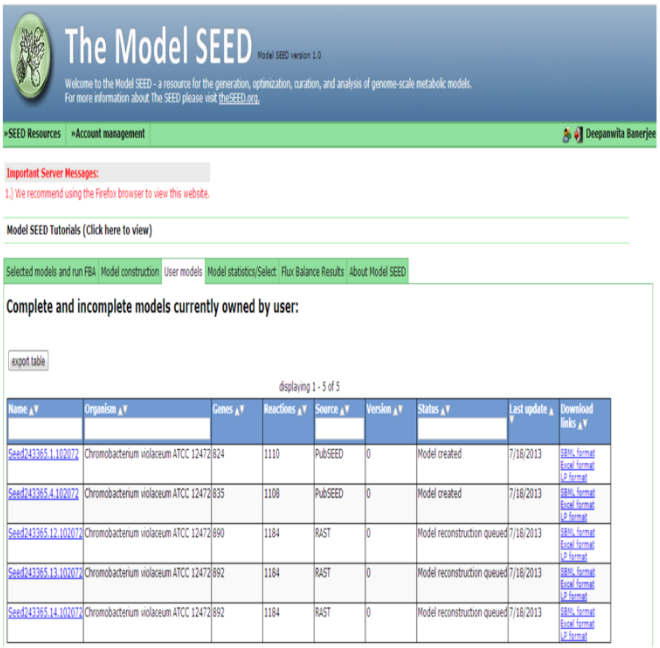

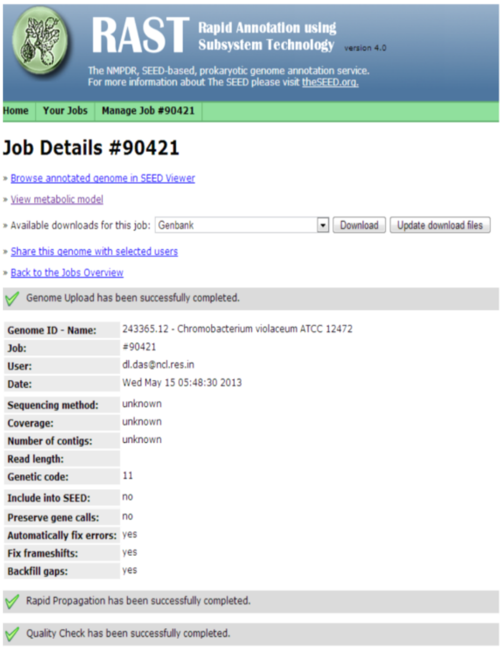

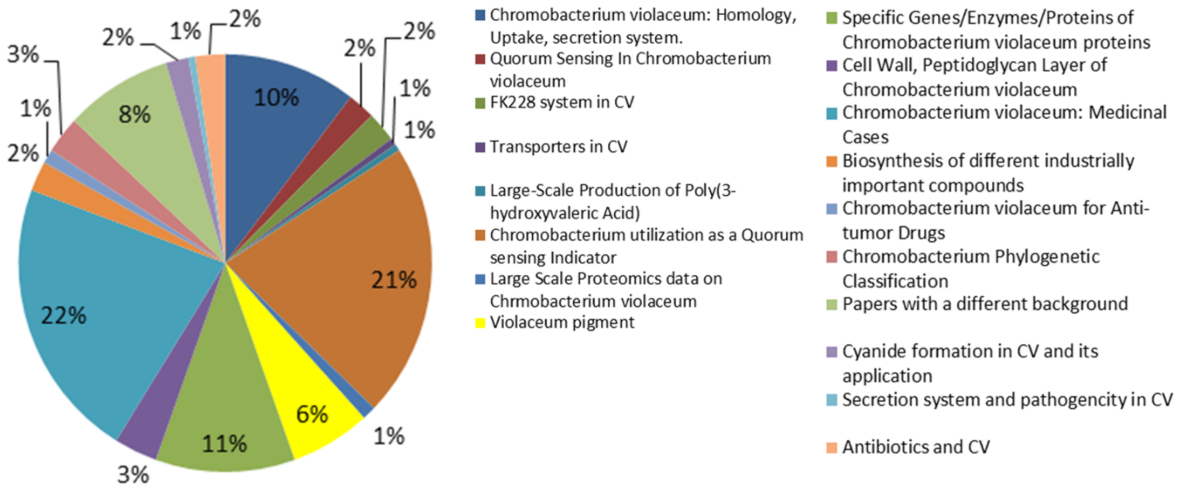

Figure B: Screen shot showing the missing chorismate mutase (red circle) in ModelSEED

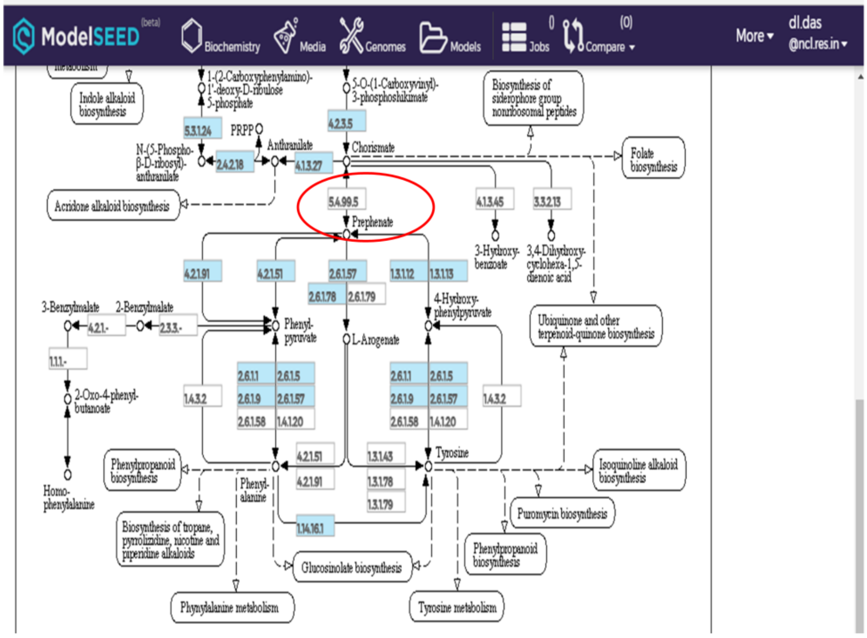

Figure C: Gene, enzyme and reaction information of chorismate mutase as in KEGG database.

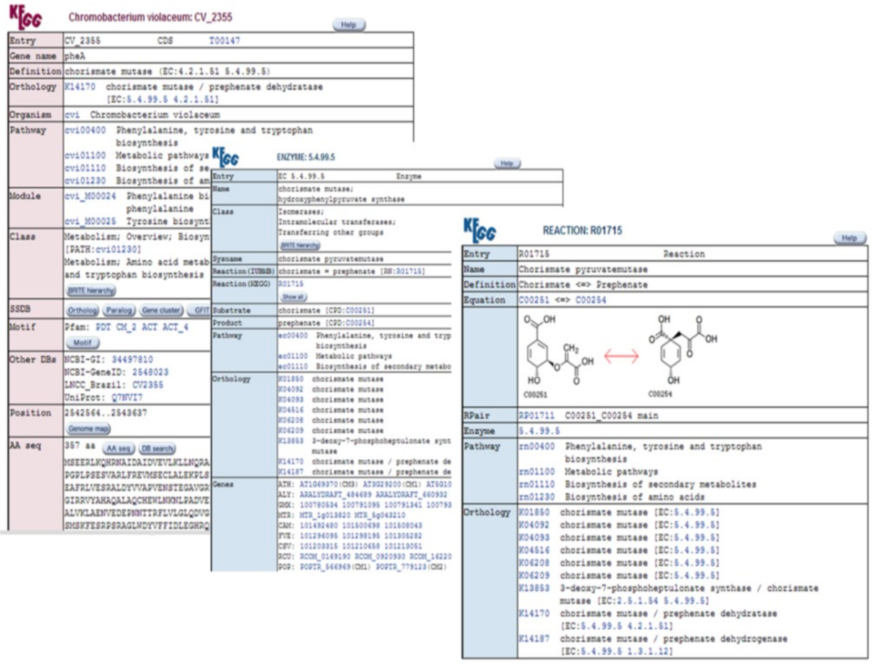

Figure D: Reaction classification for subsystems that have less than 10 reactions

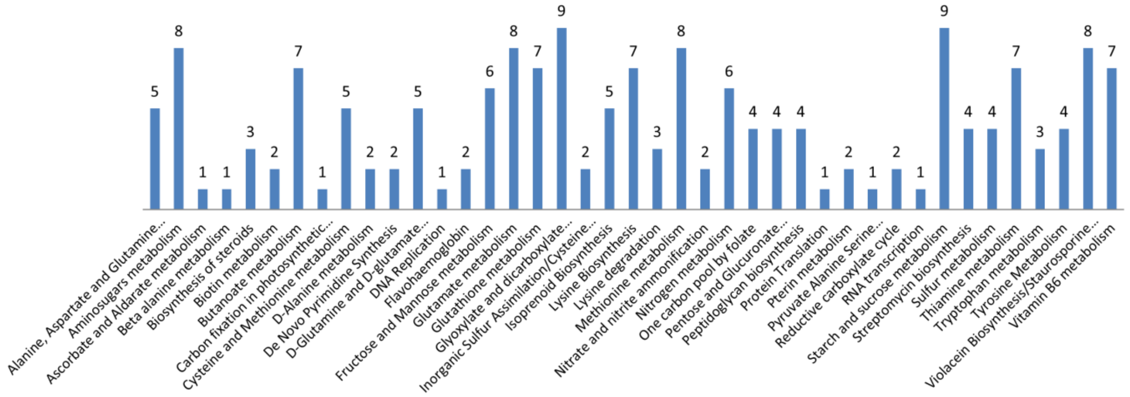

Figure E: Subsystems wise classification of essential genes common to all the substrates

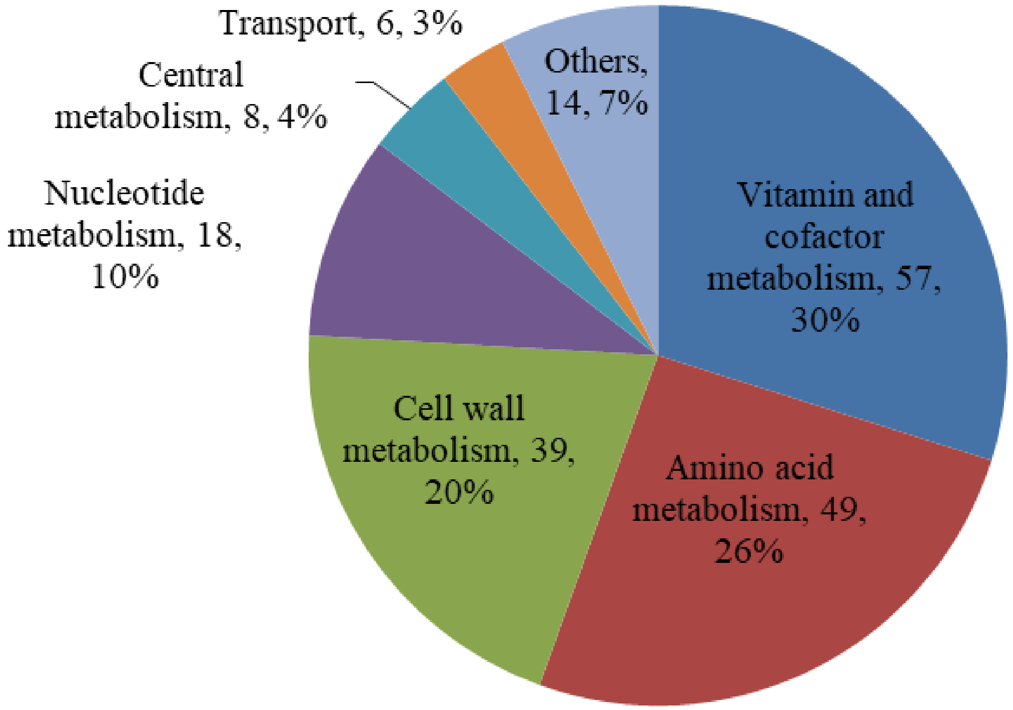

Figure F: Flux variability analysis (FVA) of WT in presence and absence of antibiotic. a) Subsystem wise reactions that change category in comparison to WT. b) Handful reactions that show change in direction compared to WT. c) Overall change in rigidity and flexibility in the three different models

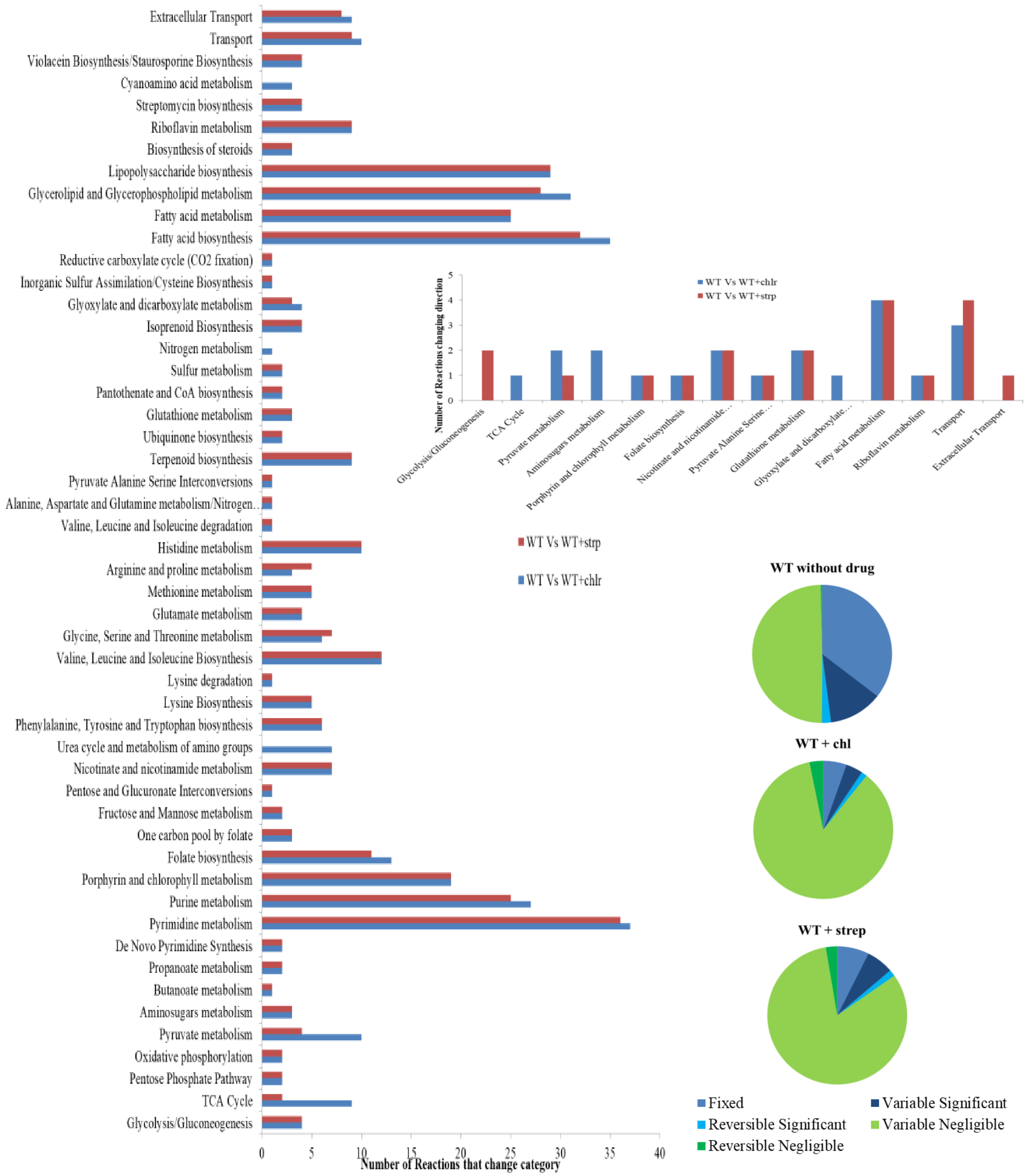

Figure G: Flux variability analysis FVA of WT and resistant population ChlR and StrpR. a) Subsystem wise reactions that change category in comparison to WT. b) Four reactions that show change in direction compared to WT. c) Overall change in rigidity and flexibility in the three different models

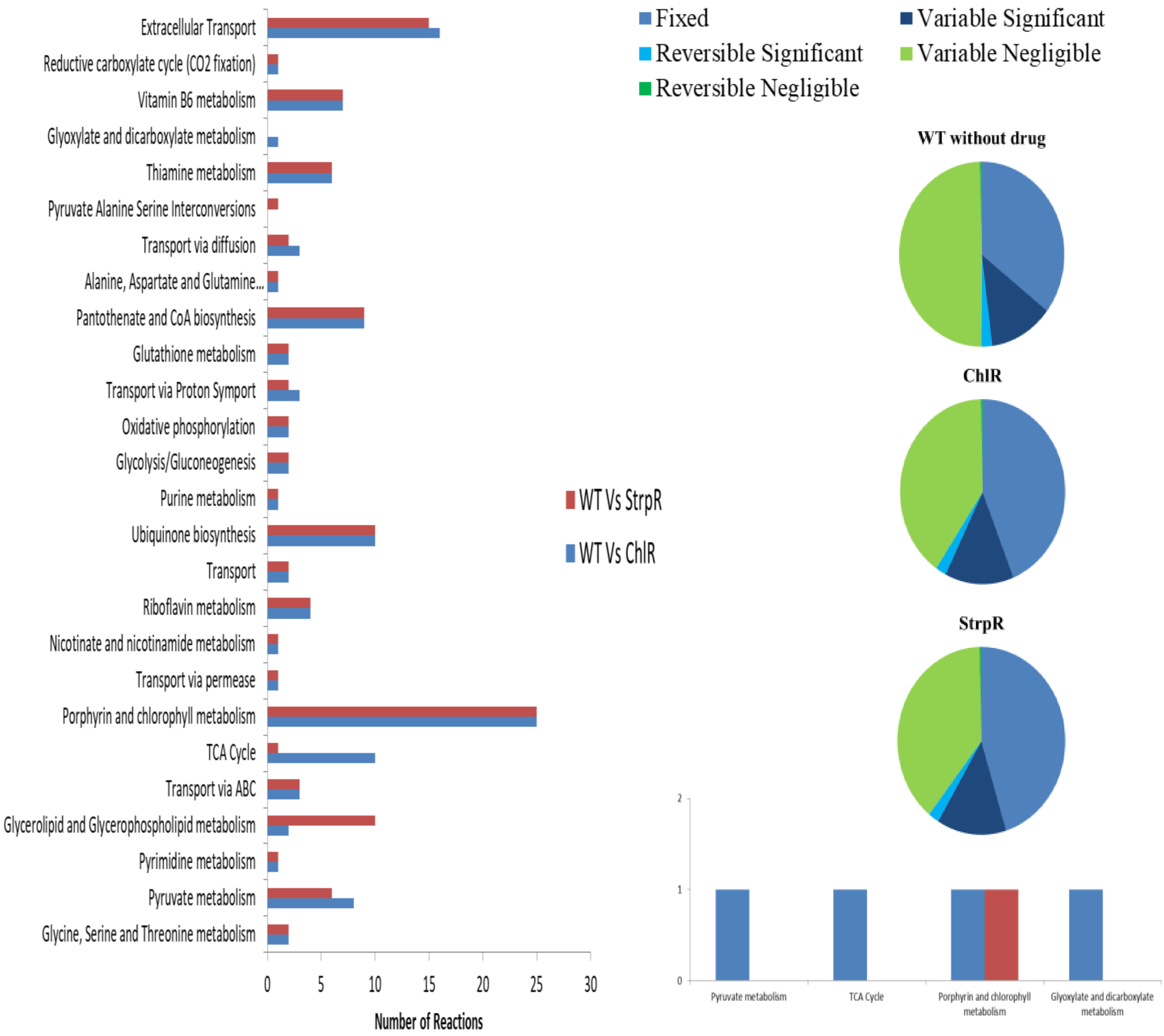

Table A: List of reactions added to *i*DB858 in order to form biomass

Reaction ID	Reaction name	GPR
rDB00002_c	Chorismate mutase/prephenate dehydratase	(CV_2355)
rDB00003_c	Acetylornithine aminotransferase	(CV_1496) or (CV_2256)
rDB00004_c	erythronate-4-phosphate dehydrogenase	(CV_3789)
rDB00005_c	(E)-4-hydroxy-3-methylbut-2-enyl-diphosphate synthase	(CV_3538)
rDB00006_c	Isoprenyl transferase	(CV_2691) or (CV_2200)
rDB00007_c	GAP filling rxn in Riboflavin metabolism	"
rDB00008_c	Acid phosphatase	(CV_3525) or (CV_4286)
rDB00009_c	Exopolyphosphatse	(CV_1262)
rDB00010_c	Non-canonical purine NTP pyrophosphatase	(CV_0926)
rDB00011_c	oxidoreductase protein	(CV_3210)
rDB00012_c	GMP synthase	(CV_3465) or (CV_3746)
rDB00013_c	Hydroxymethylpyrimidine kinase	(CV_0151)
rDB00014_c	Hydroxymethylpyrimidine kinase	(CV_0151)
rDB00015_c	Dihydrofolate reductase	(CV_1028)
rDB00016_c	formyltetrahydrofolate synthetase	(CV_1925)
rDB00017_c	phosphoribosylglycinamide formyltransferase	(CV_3616)
rDB00018_c	Aminomethyltransferase	(CV_3431)
rDB00019_c	5-methyltetrahydrofolate-homocysteine S-methyltransferase	(CV_3429) and (CV_0528 or CV_1074 or CV_2037) and (CV_3431)
rDB00020_c	glycoaldehyde dehydrogenase	"
rDB00021_c	glycolate dehydrogenase	(CV_1724)
rDB00022_c	glycerate dehydrogenase	(CV_3789 or CV_1724)
rDB00023_c	phosphoglycerate dehydrogenase	(CV_1724)
rDB00024_c	Pimeloyl-acp methyl ester esterase	"
rDB00025_c	Pimeloyl-acp methyl ester esterase	(CV_4380)
rDB00026_c	precorrin-3B synthase	"
rDB00027_c	precorrin-6A synthase	(CV_1565)
rDB00028_c	Cobalt-precorrin-7 (C5)-methyltransferase	(CV_1562)
rDB00029_c	threonine-phosphate decarboxylase	(CV_2728)
rDB00030_c	ATP: L-threonine O-phosphotransferase	(CV_1537)
rDB00031_c	Riboflavin kinase	(CV_3570)
rDB00032_c	quinolinate synthetase	(CV_3678)
rDB00033_c	nicotinamise-nucleotide amidase	(CV_2370)
rDB00034_c	NADP pyrophosphate	(CV_2905)
rDB00035_c	ATP: NMN adenyltransferase	(CV_0519)
rDB00036_c	2-oxoglutarate synthase	(CV_1071) and (CV_0528 or CV_1074 or CV_2037) and (CV_1072)
rDB00037_c	Lumped rxn for menaqunone formation	"
rDB00038_c	glutamate-cysteine ligase	(CV_4276)
rDB00039_c	Carboxynospermidine synthase	"

rDB00040_c	pyruvate decarboxylase	(CV_0586) or (CV_0587) or (CV_3889)
rDB00041_c	oleoyl-ACP hydrolase	"
rDB00042_c	Palmitoyl-CoA : electron-transfer flavoprotein 2, 3-oxidoreductase	(CV_1785) or (CV_2723) or (CV_3816)
rDB00043_c	Myristoyl-CoA : electron-transfer flavoprotein 2, 3-oxidoreductase	(CV_1785) or (CV_2723) or (CV_3816)
rDB00044_c	Lauroyl-CoA : electron-transfer flavoprotein 2, 3-oxidoreductase	(CV_1785) or (CV_2723) or (CV_3816)
rDB00045_c	Decanoyl-CoA : electron-transfer flavoprotein 2, 3-oxidoreductase	(CV_1785) or (CV_2723) or (CV_3816)
rDB00046_c	Octanoyl-CoA : electron-transfer flavoprotein 2, 3-oxidoreductase	(CV_1785) or (CV_2723) or (CV_3816)
rDB00047_c	Hexanoyl-CoA : electron-transfer flavoprotein 2, 3-oxidoreductase	(CV_1785) or (CV_2723) or (CV_3816)
rDB00048_c	N-acetyl-D-glucosamine 1-phosphate 1,6-phosphomutase	(CV_3795)
rDB00049_c	oleoyl-ACP hydrolase	"
rDB00050_c	lumped rxn for lysine biosynthesis	"
rDB00051_c	phophomethylpyrimidine synthase	(CV_0235)
rDB00052_c	Thiamine Kinase	"
rDB00053_c	GAP filling rxn in Peptidoglycan biosynthesis	(CV_0834) and (CV_2562) and (CV_3586) and (CV_4360) and (CV_4349) and (CV_1125 or CV_3094)
rDB00054_c	UDP-3-O-acyl-GlcNAc deactylase	(CV_4337)
rDB00055_c	UDP-3-O-acyl-glucosamine N-acyltransferase	(CV_2206)
rDB00056_c	UDP-2, 3-diacylglucosamine hydrolase	(CV_3186)
rDB00057_c	KDO transferase	(CV_0225)
rDB00058_c	KDO transferase	(CV_0225)
rDB00059_c	GAP filling rxn in lipopolysaccharide biosynthesis	"
rDB00060_c	D-glycero-alpha-D-manno-heptose 1, 7-bisphosphate 7-phosphatase	(CV_1657)
rDB00061_c	GAP filling rxn in lipopolysaccharide biosynthesis	"
rDB00062_c	GAP filling rxn in lipopolysaccharide biosynthesis	"
rDB00063_c	GAP filling rxn in lipopolysaccharide biosynthesis	"
rDB00064_c	GAP filling rxn in lipopolysaccharide biosynthesis	(CV_3880)
rDB00065_c	Glycosyltransferase	(CV_0817)
rDB00066_c	GAP filling rxn in lipopolysaccharide biosynthesis	"
rDB00067_c	Hydrogenobyrinate-acid-a, c-diamide : cobalt cobalt-ligase	(CV_1571)
rDB00068_c	Oxygen insensitive nadph nitro reductase/ FMN reductase	(CV_3500)
rDB00069_c	Sink needed for 4-Hydroxy-benzylalcohol to leave system	"
rDB00133_c	5,6-dimethylbenzimidazole synthase	CV_1555

Table B: Biomass composition of *C. violaceum*

Component	% Dry Weight	Organism
Protein[1]	41.33	*N.meningitidis*
RNA[2]	17.64	*C.violaceum*
DNA[3]	7.57	*C.violaceum*
Phospholipids[1]	6.64	*N.meningitidis*
Peptidoglycan[3]	0.06	*C.violaceum*
Lipopolysaccharide[3]	4.42	*C.violaceum*
Putrescine[4]	0.231	*C.violaceum*
Spermidine[4]	0.003	*C.violaceum*

Table C: Biomass equation of *i*DB858

	Mets	Type of Met		Coefficient
1	thr__L_c	AA	L-Threonine	0.16293
2	gly_c	AA	Glycine	0.31790
3	atp_c	NUC	ATP	59.81000
4	dctp_c	NUC	dCTP	0.07863
5	coa_c	VIT&CO	Coenzyme A	0.003097
6	arg__L_c	AA	L-Arginine	0.26227
7	h2o_c	Ions	H2O	59.81000
8	nad_c	VIT&CO	NAD	0.003097
9	dttp_c	NUC	dTTP	0.04423
10	met__L_c	AA	L-Methionine	0.09140
11	ala__L_c	AA	L-Alanine	0.47288
12	nadp_c	VIT&CO	NADP	0.003097
13	val__L_c	AA	L-Valine	0.25432
14	mg2_c	Ions	Magnesium	0.003097
15	mqn8_c	VIT&CO	Menaquinone 8	0.003097
16	gtp_c	NUC	GTP	0.17558
17	ribflv_c	VIT&CO	Riboflavin	0.003097
18	glu__L_c	AA	L-Glutamate	0.20266
19	dgtp_c	NUC	dGTP	0.07863
20	lys__L_c	AA	L-Lysine	0.13511
21	asp__L_c	AA	L-Aspartate	0.20664
22	ser__L_c	AA	L-Serine	0.21458
23	sheme_c	VIT&CO	Siroheme	0.003097
24	fe2_c	Ions	Fe2+	0.003097
25	ctp_c	NUC	CTP	0.17558
26	pro__L_c	AA	L-Proline	0.18677
27	k_c	Ions	Potassium	0.003097
28	zn2_c	Ions	Zinc	0.003097
29	udcpdp_c	PEPTIDO	Bactoprenyl diphosphate	0.00059

30	peptido_CV_c	PEPTIDO	Peptidoglycan polymer (n subunits)	0.00059
31	cys__L_c	AA	L-Cysteine	0.03974
32	so4_c	Ions	Sulfate	0.003097
33	q8_c	VIT&CO	Ubiquinone-8	0.003097
34	gln__L_c	AA	L-Glutamine	0.16690
35	datp_c	NUC	dATP	0.04423
36	gthrd_c	VIT&CO	GSH	0.003097
37	spmd_c	POLYNH2	Spermidine	0.00030
38	leu__L_c	AA	L-Leucine	0.43712
39	tyr__L_c	AA	L-Tyrosine	0.09537
40	thf_c	VIT&CO	5,6,7,8-Tetrahydrofolate	0.003097
41	his__L_c	AA	L-Histidine	0.08345
42	fad_c	VIT&CO	Flavin adenine dinucleotide oxidized	0.003097
43	amet_c	VIT&CO	S-Adenosyl-L-methionine	0.003097
44	5mthf_c	VIT&CO	5-Methyltetrahydrofolate	0.003097
45	utp_c	NUC	UTP	0.09876
46	10fthf_c	VIT&CO	10-Formyltetrahydrofolate	0.003097
47	cobalt2_c	Ions	Co2+	0.003097
48	asn__L_c	AA	L-Asparagine	0.10729
49	ptrc_c	POLYNH2	Putrescine	0.04410
50	trp__L_c	AA	L-Tryptophan	0.05563
51	pydx5p_c	VIT&CO	Pyridoxal 5-phosphate	0.003097
52	phe__L_c	AA	L-Phenylalanine	0.13114
53	adocbl_c	VIT&CO	Adenosylcobalamin	0.003097
54	thmpp_c	VIT&CO	Thiamine diphosphate	0.003097
55	ile__L_c	AA	L-Isoleucine	0.16690
56	pheme_c	VIT&CO	Protoheme	0.003097
57	2dmmq8_c	VIT&CO	2-Demethylmenaquinone 8	0.003097
58	fe3_c	Ions	Fe3+	0.003097
59	cl_c	Ions	Chloride	0.003097
60	mn2_c	Ions	Mn2+	0.003097
61	cu2_c	Ions	Cu2+	0.003097
62	ca2_c	Ions	Calcium	0.003097
63	colipa_c	LPS	core oligosaccharide lipid A	0.02321
64	pe120_c	PLIPID	Dodecanoylphosphatidylethanolamine	0.00452
65	pe140_c	PLIPID	Tetradecanoylphosphatidylethanolamine	0.00137
66	pe160_c	PLIPID	Phosphatidylethanolamine_dihexadecanoyl	0.01927
67	pe161_c	PLIPID	Phosphatidylethanolamine_dihexadec-9enoyl	0.02886
68	pe181_c	PLIPID	Phosphatidylethanolamine_dioctadec-11-enoyl	0.01209
69	pe170cyc_c	PLIPID	pe170cyc	0.00137
70	pg120_c	PLIPID	Phosphatidylglycerol_didodecanoyl	0.00106
71	pg140_c	PLIPID	Phosphatidylglycerol_ditetradecanoyl	0.00032
72	pg160_c	PLIPID	Phosphatidylglycerol_dihexadecanoyl	0.00450
73	pg161_c	PLIPID	Phosphatidylglycerol_dihexadec-9-enoyl	0.00675
74	pg181_c	PLIPID	Phosphatidylglycerol_dioctadec-11-enoyl	0.00283
75	pg170cyc_c	PLIPID	pg170cyc	0.00032
76	clpn120_c	PLIPID	clpn120	0.00027
77	cpd15792_c	PLIPID	clpn140	0.00008
78	cpd15791_c	PLIPID	clpn160	0.00116
79	clpn161_c	PLIPID	clpn161	0.00173

80	clpn181_c	PLIPID	clpn181	0.00073
81	clpn170cyc_c	PLIPID	clpn170cyc	0.00008
1	ppi_c	Ions	Diphosphate	0.4846
2	h_c	Ions	H+	59.81000
3	adp_c	NUC	ADP	59.81000
4	pi_c	Ions	Phosphate	59.81000

Table D: Reaction information for the cyanide biosynthesis module

Reaction ID	Reaction Name	KEGG RID	Reaction Formula
rDB00166_c	glycine:acceptor oxidoreductase	R05704	gly_c + 2 nadph_c -> co2_c + 2 nadp_c + hcn_c
rDB00167_c	cyn_rxn2	R06614	HC00955_c <=> h2o_c + 3Aprop_c
rDB00168_c	γ-Amino-γ-cyanobutanoate aminohydrolase/nitrilase	R01887	2 h2o_c + acybut_c <=> glu__L_c + nh4_c
rDB00169_c	α-Aminopropiononitrile aminohydrolase/Nitrilase	R03542	2 h2o_c + aprop_c <=> ala__L_c + nh4_c
rDB00170_c	cyn_rxn5	R01410	hcn_c -> aprop_c
rDB00171_c	cyn_rxn6	R01650	hcn_c -> acybut_c
rDB00172_c	cyn_rxn7	R03524	cys__L_c + hcn_c -> HC00955_c
rDB00173_c	cyn_rxn8	R01267	HC00955_c -> asn__L_c
rxn02792_c	(5-Glutamyl)-peptide:amino-acid 5-glutamyltransferase	R03971	h_c + glu__L_c + 3Aprop_c -> co2_c + h2o_c + HC01700_c
rxn02791_c	(5-Glutamyl)-peptide:amino-acid 5-glutamyltransferase	R03970	glu__L_c + HC00955_c <=> h2o_c + HC01577_c

Table E: Reaction information for the violacein biosynthesis module

Reaction ID	Reaction Name	KEGGID	Reaction Formula
rDB00091_c	Tryptophan 2-monooxygenase	R11119	o2_c + trp__L_c -> h_c + h2o2_c + mDB_2i3ip_c
rDB00092_c	PDVnate synthesis	R11131	2 mDB_2i3ip_c -> co2_c + nh4_c + mDB_pdvnate_c
rDB00093_c	Protodeoxyviolaceinate, NADPH:o2 oxidoreductase	R11134	h_c + nadph_c + o2_c + mDB_pdvnate_c -> h2o_c + nadp_c + mDB_pvnate_c
rDB00094_c	Protoviolaceinate, NADPH:o2 oxidoreductase	R11135	h_c + nadph_c + o2_c + mDB_pvnate_c -> h2o_c + nadp_c + mDB_vnate_c
rDB00095_c	Violacein spontaneous synthesis	R11136	h_c + o2_c + mDB_vnate_c -> co2_c + h2o_c + mDB_vio_c
rDB00096_c	Protodeoxyviolaceinate, NADPH:o2 oxidoreductase	R11374	h_c + nadph_c + o2_c + mDB_pdvnate_c -> h2o_c + nadp_c + mDB_dvnate_c
rDB00097_c	Deoxyviolacein spontaneous synthesis	R11133	h_c + o2_c + mDB_dvnate_c -> co2_c + h2o_c + mDB_dvio_c
rDB00098_c	Prodeoxyviolacein spontaneous synthesis	None	h_c + o2_c + mDB_pdvnate_c -> co2_c + h2o_c + mDB_prodvio_c
rDB00099_c	Diffusion of violacein	None	h_c + mDB_vio_c -> h_e + mDB_vio_e
rDB00100_c	Diffusion of prodeoxyviolacein	None	mDB_prodvio_c -> mDB_prodvio_e

rDB00101_c	Diffusion of deoxyviolacein	None	h_c + mDB_dvio_c -> h_e + mDB_dvio_e
EX_vio_e	Violacein Exchange	None	mDB_vio_e <=>
EX_dvio_e	Deoxyviolacein Exchange	None	mDB_dvio_e <=>
EX_prodvio_e	Prodeoxyviolacein Exchange	None	mDB_prodvio_e <=>

Table F: *In silico* prediction for the 57 BIOLOG GN2 plate substsrates

Substrate	Lima-Bittencourt et al., 2011	Young et al., 2008	Martin et al., 2007	*i*DB858	Biomass
Sucrose	+		+	+	1.973
D-mannose	+	+		+	0.987
D-trehalose	+	+		+	1.973
L-phenylalanine	+	+		+	0.922
L-threonine	+	+		+	0.608
Inosine	+	+		+	0.689
Thymidine	+	+		+	0.888
D, L-a-glycerol phosphate	+	+		+	0.574
D-glucose-6-phosphate	+	+		+	1.051
N-acetyl-D-glucosamine	+			+	1.169
D-cellobiose	+			+	1.973
D-fructose	+			+	0.981
a-D-glucose	+			+	0.987
D, L-lactic acid	+			+	0.413
Succinic acid	+			+	0.472
D-alanine	+			+	0.383
L-alanine	+			+	0.440
L-alanylglycine	+			+	0.646
L-asparagine	+			+	0.467
L-aspartic acid	+			+	0.440
L-glutamic acid	+			+	0.432
Glycyl-L aspartic acid	+			+	0.675
Glycyl-L glutamic acid	+			+	0.657
L-histidine	+			+	0.440
a-Cyclodextrin	+			+	5.920
Dextrin	+			+	5.920
Glycogen	+			+	3.946
L-arabinose	+			+	0.614
D-arabitol	+			+	0.682
D-psicose	+			+	0.987
Turanose	+			+	1.973
L-ornithine	+			+	0.608

L-proline	+			+	0.564
L-serine	+			+	0.374
D-gluconic acid	+			+	0.860
b-Hydroxybutyric acid	+			+	0.525
Urocanic acid	+			+	0.485
D-serine	-	+		+	0.348
Uridine	-	+		+	0.679
2-Aminoethanol	-	+		+	0.334
Cis-aconitic acid	+	-		+	0.656
Formic acid*	-			+	0.054
Glycerol*	-			+	0.571
L-leucine#	+	-		-	0.000
Citric acid	-	-	+	-	0.000
a-D-glucose-1-phosphate#	-	+		-	0.000
a-Keto butyric acid	-			-	0.000
a-Keto glutaric acid	-	-		-	0.000
c-Amino butyric acid	-	-		-	0.000
Putrescine	-			-	0.000
D-galactose	-			-	0.000
m-Inositol	-			-	0.000
Maltose	-			-	0.000
D-mannitol	-			-	0.000
Acetic acid	-		-	-	0.000
D-sorbitol	-			-	0.000
Propionic acid	-			-	0.000

* Experimental evidence exists, # Conflicting literature evidence

Table G: In silico prediction accuracy of *i*DB858 for the 29 Ex-mets

Mets	Growth	Violacein	Cyanide	% Prediction Accuracy
Glc	0.967	0.0007	0.006	62.9
g6p	1.033	0.0007	0.006	95.6
g3p	0	0.0009	0.000	100.0
Fdp	1.082	0.0005	0.005	94.9
Fum	0.853	0.0005	0.005	54.9
Male	0.421	0.0009	0.009	69.8
D-Mal	0.827	0.0006	0.006	77.2
Succ	0.920	0.0008	0.008	91.9
2oxoadp	0.704	0.0022	0.021	85.6
MLO	0.561	0.0007	0.007	52.1
Pyr	0.667	0.0004	0.004	84.3
Cit	0.000	0.0005	0.000	100.0

Icit	1.292	0.0000	0.000	98.6
Lact	0.827	0.0007	0.007	83.4
Kga	0.258	0.0005	0.005	62.5
Ara	0.587	0.0004	0.004	66.5
m6p	1.033	0.0007	0.006	95.9
r5p	0.652	0.0007	0.007	75.5
3pg	0.397	0.0005	0.005	60.3
Trp	0.000	0.0007	0.000	0.0
Ala	0.419	0.0008	0.015	43.8
Val	0.000	0.0007	0.000	0.0
Asp	0.208	0.0007	0.013	89.2
Gln	0.000	0.0007	0.000	0.0
Glu	0.389	0.0005	0.009	30.4
Man	0.000	0.0006	0.000	0.0
Sbt	0.000	0.0007	0.000	0.0
Glyc	0.544	0.0006	0.006	69.8
Ascb	0.570	0.0006	0.006	70.4

Table H: Experimental condition used to define wild type growth on different substrates

Substrate	Model	VSR*	ATPM#	Molar growth yield*	Oxygen uptake rate#
Pyruvate	WT	0.013	3.62	0.0092	1.927
Succinate	WT	0.019	4.94	0.0117	2.7222
D-malate	WT	0.027	2.6	0.0153	1.9392

* Experimental values # determined *in silico*

Table I: Experimental condition used to define resistant population growth on different substrates

Substrate	Model	VSR*	Molar growth yield*	Time of growth (hr)	ATPM#	*In silico* Molar growth yield#	Oxygen uptake rate#
Pyruvate	ChlR	0.013	0.0182	After 24	5.3	0.00027	2.2308
	StrpR	0.013	0.0157	<3	3.36	0.0106	1.8797
Succinate	ChlR	0.019	0.0160	<3	7.57	0	0
	StrpR	0.019	0.0150	<3	4.73	0.0128	2.6849
D-malate	ChlR	0.027	0.0282	0 – 24	3.99	0.0079	2.19
	StrpR	0.027	0.0205	<3	2.53	0.0157	1.9263

* Experimental values # determined *in silico*

Table J: Confidence score assigned during manual curation of the reconstruction obtained from SEED server

Score	Evidence	Type of Evidence
1.1	Gene is not present in CV but reaction essential for biomass production. Gene with known function is present in unrelated organism.	Homology in phylogenetically un-related organism
1.2	Enzyme not present in CV but indirect biochemical evidence of existence of the reaction is present. <30%.	Homology
1.3	Inferred from homology (as per UNIPROT data). Gene present in CV but very low match with other organism. 30% < 1.3 < 60%.	Homology
1.4	Gene present in CV and reaction essential for biomass production. Gene with known function present in related organism.	Homology in phylogenetically related organism
1.5	Gene present in CV and reaction essential for biomass production. Gene with known function present in well-known organism – *E. coli, Neisseria, franscisella, Pseudomonas, Ralstonia*, etc. >60%	Homology
1.6	Homology based evidence with a sequence homology =>95% with other protein with known crystal structure, function or biochemical evidence	Homology based Biochemical evidence
2.1	Physiological (phenotypic) evidence of utilization/uptake of the protein in CV (different structure growth, BIOLOG, etc.)	Physiological
2.2	Protein has been identified based on MS/ LC-MS/ MALDI. Evidence of uptake or utilization of compound, phenotypic evidence	Physiological
3.1	Cloning, expression and over-expression	Genetic evidence
3.2	Gene deletion studies	Genetic evidence
4.1	Biochemical evidence in very closely related species of CV. Ex: CV026.	Biochemical
4.2	Enzyme present and evidence of the reaction catalyzed in CV	Biochemical

Table K: Selected reactions showing changes in FVA of WT in absence of antibiotic (WT) and presence of chloramphenicol (WT+chl) for *C. violaceum*

SUBSYSTEM	BIGGID	CHEMICAL FORMULA	WT	WT +chl	
Glycolysis or Gluconeogenesis	HEX1	atp_c + glc__D_c -> adp_c + g6p_c	1	7d	
	PYK	adp_c + pep_c -> atp_c + pyr_c	7d	1	
TCA Cycle	AKGDH	coa_c + nad_c + akg_c -> co2_c + nadh_c + succoa_c	3	7d	
	FRD7	succ_c + q8_c <=> fum_c + q8h2_c	2	7c	
	FUM	mal__L_c <=> fum_c + h2o_c	4	7b	
	MDH	nad_c + mal__L_c <=> h_c + nadh_c + oaa_c	2	7a	
	CS	accoa_c + h2o_c + oaa_c -> h_c + coa_c + cit_c	1	7d	
Oxidative phosphorylation	cytochrome oxidase bo3 ubiquinol-8 2.5 protons	2.5 h_c + 0.5 o2_c + q8h2_c -> h2o_c + 2.5 h_e + q8_c	2	1	0.08
Pyruvate metabolism	PFL	accoa_c + for_c <=> coa_c + pyr_c	8	5	0.1
	MALS	accoa_c + h2o_c + glx_c -> h_c + coa_c + mal__L_c	1	7d	
	ME2	nadp_c + mal__L_c -> co2_c + nadph_c + pyr_c	3	7d	
	PTAr	h_c + accoa_c + pi_c -> actp_c + coa_c	7d	1	
	ACKr	actp_c + adp_c -> h_c + atp_c + ac_c	7d	1	
	OAADC	h_c + oaa_c -> co2_c + pyr_c	3	7d	
Purine metabolism	ADK1	atp_c + amp_c -> 2 adp_c	2	1	0.0005
	ATP carbamate phosphotransferase	atp_c + co2_c + nh4_c <=> h_c + adp_c + cbp_c	1	7a	
	NDPK8	atp_c + dadp_c -> adp_c + datp_c	3	7d	
Pyrimidine metabolism	NDPK2	atp_c + udp_c -> adp_c + utp_c	2	7a	
Folate biosynthesis	MTHFD	nadp_c + mlthf_c <=> nadph_c + methf_c	1	7a	
	FTHFD	h2o_c + 10fthf_c -> h_c + for_c + thf_c	1	7a	
Glutamate metabolism	ASPTA	asp__L_c + akg_c <=> oaa_c + glu__L_c	4	7b	
Glycine, Serine and Threonine metabolism	PSERT	akg_c + pser__L_c <=> glu__L_c + 3php_c	4	4	0.0005
	GHMT	gly_c + h2o_c + mlthf_c <=> ser__L_c + thf_c	4	7b	
Arginine and proline metabolism	PRO1x	h_c + nadh_c + 1pyr5c_c -> nad_c + pro__L_c	3	7d	
	P5CR	h_c + nadph_c + 1pyr5c_c -> nadp_c + pro__L_c	3	7d	
Glyoxylate and dicarboxylate metabolism	ICL	icit_c <=> succ_c + glx_c	1	7c	
Nitrogen metabolism	Carbonic acid hydro-lyase	co2_c + h2o_c -> h_c + hco3_c	1	7a	
Urea cycle and metabolism of amino groups	ARGSL	argsuc_c -> fum_c + arg__L_c	1	7a	
	ARGSS_1	atp_c + asp__L_c + citr__L_c -> ppi_c + argsuc_c + amp_c	1	7a	
	AGGPR	nadph_c + acg5p_c -> pi_c + nadp_c + acg5sa_c	1	7a	
	OCBT	cbp_c + orn_c -> 2 h_c + pi_c + citr__L_c	1	7a	
	ORNTAC	glu__L_c + acorn_c <=> orn_c + acglu_c	1	7a	

Cyanoamino Metabolism	ACGK	h_c + atp_c + acglu_c -> adp_c + acg5p_c	1	7a
	ACOTA	glu__L_c + acg5sa_c -> akg_c + acorn_c	1	7a
	glycine:acceptor oxidoreductase	gly_c + 2 nadph_c -> co2_c + 2 nadp_c + hcn_c	1	7a
	γ-Amino-γ-cyanobutanoate aminohydrolase/nitrilase	2 h2o_c + acybut_c <=> glu__L_c + nh4_c	1	7a
	cyn_rxn6	hcn_c -> acybut_c	1	7a
Glycerolipid and Glycerophospholipid metabolism	G3PD	glyc3p_c + fad_c -> dhap_c + fadh2_c	3	7d
	sn-Glycerol-3-phosphate NADP 2-oxidoreductase	h_c + nadph_c + dhap_c -> glyc3p_c + nadp_c	3	7d
	sn-Glycerol-3-phosphate NAD 2-oxidoreductase	h_c + nadh_c + dhap_c -> nad_c + glyc3p_c	3	7d
Transport via ABC	Orthophosphate-ABC transport	atp_c + h2o_c + pi_e -> h_c + adp_c + 2 pi_c	1	7a
Extracellular Transport	Exchange	for_e <=>	7d	1
	EX_ac_e	ac_e <=>	7d	1

Table L: Selected reactions showing changes in FVA of WT in absence of antibiotic (WT) and presence of streptomycin (WT+strep) for *C. violaceum*

SUBSYSTEM	BIGGID	CHEMICAL FORMULA	WT	WT+ strep	
TCA Cycle	AKGDH	coa_c + nad_c + akg_c -> co2_c + nadh_c + succoa_c	3	3	20.73
	FUM	mal__L_c <=> fum_c + h2o_c	4	5	0.59
	MDH	nad_c + mal__L_c <=> h_c + nadh_c + oaa_c	2	2	0.36
Oxidative phosphorylation	cytochrome oxidase bo3 ubiquinol-8 2.5 protons	2.5 h_c + 0.5 o2_c + q8h2_c -> h2o_c + 2.5 h_e + q8_c	2	1	0.32
Pyruvate metabolism	PFL	accoa_c + for_c <=> coa_c + pyr_c	8	8	0.27
	PPS	atp_c + h2o_c + pyr_c -> h_c + pi_c + pep_c + amp_c	7d	1	
Purine metabolism	ATP carbamate phosphotransferase	atp_c + co2_c + nh4_c <=> h_c + adp_c + cbp_c	1	1	6.63
Folate biosynthesis	MTHFD	nadp_c + mlthf_c <=> nadph_c + methf_c	1	1	2.67
	FTHFD	h2o_c + 10fthf_c -> h_c + for_c + thf_c	1	1	5
Glutamate metabolism	ASPTA	asp__L_c + akg_c <=> oaa_c + glu__L_c	4	5	1.75
Glycine, Serine and Threonine metabolism	PSERT	akg_c + pser__L_c <=> glu__L_c + 3php_c	4	5	0.31
	GHMT	gly_c + h2o_c + mlthf_c <=> ser__L_c + thf_c	4	5	2.39
Arginine and proline	PRO1x	h_c + nadh_c + 1pyr5c_c -> nad_c +	3	3	0.005

metabolism		pro__L_c			
	SOTA	akg_c + sucorn_c <=> sucgsa_c + glu__L_c	7d	1	
	SGSAD	h2o_c + nad_c + sucgsa_c -> 2 h_c + nadh_c + sucglu_c	7d	1	
	SGDS	h2o_c + sucglu_c <=> succ_c + glu__L_c	7d	1	
	AST	arg__L_c + succoa_c -> h_c + coa_c + sucarg_c	7d	1	
	N2-succinyl-L-arginine iminohydrolase decarboxylating	2 h_c + 2 h2o_c + sucarg_c -> co2_c + sucorn_c + 2 nh4_c	7d	1	
Urea cycle and metabolism of amino groups	ARGSL	argsuc_c -> fum_c + arg__L_c	1	1	16.65
	ARGSS_1	atp_c + asp__L_c + citr__L_c -> ppi_c + argsuc_c + amp_c	1	1	16.65
	AGGPR	nadph_c + acg5p_c -> pi_c + nadp_c + acg5sa_c	1	1	14.25
	OCBT	cbp_c + orn_c -> 2 h_c + pi_c + citr__L_c	1	1	16.65
	ORNTAC	glu__L_c + acorn_c <=> orn_c + acglu_c	1	1	14.26
	ACGK	h_c + atp_c + acglu_c -> adp_c + acg5p_c	1	1	14.25
	ACOTA	glu__L_c + acg5sa_c -> akg_c + acorn_c	1	1	14.25
Cyanoamino Metabolism	glycine:acceptor oxidoreductase	gly_c + 2 nadph_c -> co2_c + 2 nadp_c + hcn_c	1	1	4
	cyn_rxn6	hcn_c -> acybut_c	1	1	4
			1	1	4
Extracellular Transport	NH4+ Exchange	nh4_e <=>	4	1	

Table M: Selected reactions from Flux variability analysis for WT, ChlR and StrpR at experimental constraints

Subsystem	Reaction ID	Reaction Formula	WT	ChlR		StrpR	
				Cat	Fold change	Cat	Fold change
Glycolysis or Gluconeogenesis	HEX1	atp_c + glc__D_c -> adp_c + g6p_c	1	7d		7d	
	PYK	adp_c + pep_c -> atp_c + pyr_c	7d	1		1	
TCA Cycle	AKGDH	coa_c + nad_c + akg_c -> co2_c + nadh_c + succoa_c	3	7d		3	2.56
	SUCOAS	atp_c + coa_c + succ_c -> adp_c + pi_c + succoa_c	7d	1		7d	
	FRD7	succ_c + q8_c <=> fum_c + q8h2_c	2	8		2	0.12
	MDH	nad_c + mal__L_c <=> h_c + nadh_c + oaa_c	2	1	0.04	1	0.28
	CS	accoa_c + h2o_c + oaa_c -> h_c + coa_c + cit_c	1	7d		1	0.1
	ICDHyrb	nadp_c + icit_c <=> h_c + mDB_oxasucc_c + nadph_c	7d	1		7d	
	ICDHyra	h_c + mDB_oxasucc_c -> co2_c + akg_c	7d	1		7d	

Oxidative phosphorylation	cytochrome oxidase bo3 ubiquinol-8	2.5 h_c + 0.5 o2_c + q8h2_c -> h2o_c + 2.5 h_e + q8_c	2	1	0.46	1	0.91
	cytochrome oxidase bd menaquinol-8	2 h_c + mql8_c + 0.5 o2_c -> h2o_c + mqn8_c + 2 h_e	3	7d		7d	
Pyruvate metabolism	PTAr	h_c + accoa_c + pi_c -> actp_c + coa_c	7d	1		1	
	ACKr	actp_c + adp_c -> h_c + atp_c + ac_c	7d	1		1	
	ACALD	acald_c + coa_c + nad_c <=> h_c + accoa_c + nadh_c	7a	2		1	
	PFL	accoa_c + for_c <=> coa_c + pyr_c	8	5	0.27	8	1.37
	MALS	accoa_c + h2o_c + glx_c -> h_c + coa_c + mal__L_c	1	7d		1	0.1
	ME2	nadp_c + mal__L_c -> co2_c + nadph_c + pyr_c	3	7d		7d	
	PPC	co2_c + h2o_c + pep_c -> 2 h_c + pi_c + oaa_c	7d	1		1	
	OAADC	h_c + oaa_c -> co2_c + pyr_c	3	7d		7d	
Pyruvate Alanine Serine Interconversions	D-Amino acid dehydrogenase	h2o_c + fad_c + ala__D_c <=> pyr_c + nh4_c + fadh2_c	7b	7b		4	
Glyoxylate and dicarboxylate metabolism	ICL	icit_c <=> succ_c + glx_c	1	4		1	0.1
Glutathione metabolism	AMPTASE CG	h2o_c + cgly_c <=> gly_c + cys__L_c	7b	5		4	
	glutathione hydralase	h2o_c + gthrd_c <=> glu__L_c + cgly_c	7b	5		4	
Purine metabolism	ADK2	h_c + amp_c + pppi_c -> ppi_c + adp_c	7a	2		1	
Pyrimidine metabolism	CYTK1	atp_c + cmp_c -> adp_c + cdp_c	1	2	1.3	2	2.56
Porphyrin and chlorophyll metabolism	FeII oxygen oxidoreductase	4 h_c + o2_c + 4 fe2_c <=> 2 h2o_c + 4 fe3_c	7a	7b		4	
Reductive carboxylate cycle (CO2 fixation)	ACS	h_c + atp_c + ac_c + coa_c -> ppi_c + accoa_c + amp_c	1	7d		7d	
Extracellular Transport	EX_ac_e	ac_e <=>	7d	1		1	
	EX_for_e	for_e <=>	7d	1		7d	

Table N: Category change in FVA analysis before and after adding NADH oxidase to the resistant population models

Models Compared	No. Of Reactions

	changed Category
WT VS ChlR#	123
WT VS StrpR#	117
WT VS ChlR@	122
WT VS StrpR@	117
WT VS ChlRNox#	106
WT VS StrpRNox#	120
WT VS ChlRNox@	106
WT VS StrpRNox@	117
ChlR# VS ChlRNox#	25
StrpR# VS StrpRNox#	13
ChlR# VS ChlRnox@	23
StrpR# VS StrpRNox@	8
ChlR# VS ChlR@	25
StrpR# VS StrpR@	0

# – using their respective ATPM values, @ – using the WT model ATPM

Table O: *C. violaceum* infection cases in India

	Year	Location	Age/Sex	Clinical presentation	Antibiotics given	Outcome	Reference
1	1979	Andhra Pradesh	4/M	Septicemia Meningitis		Fatal	Annapurna et al., 1979
2	1987	Karnataka	NB/M	Meningitis		Fatal	Shetty et al., 1987
3	2000	Karnataka	2yr 10 months/F	Diarrhea		Recovered	Ballal et al., 2000
4	2002	Karnataka	2months/F	Pustules, Ear Discharge, Septicemia and Meningitis		Fatal	Chattopadhyay et al., 2002
5	2002	Karnataka	8 days	Pustules, Septicemia, Meningitis and Multiple abscesses		Fatal	Shenoy et al., 2002
6	2002	Chandigarh	6.5/M	Septicemia, Pustules		Recovered	Ray et al., 2004
7	2003	West Bengal	24/M	Abscess Leg		Recovered	Dutta et al., 2003
8	2008	Kerala	6months/M	Septicemia, Skin Pustules, Broncho pneumonia		Recovered	Vijayan et al., 2009
9	2012	Vellore	40/M		PTZ later changed to meropenem	Fatal	Karthik et al., 2012

10	2012	Mumbai	11/F	Multiple liver & splenic abscesses with skin lesions & cardiogenic shock	PTZ and Gent; Ciprofloxacin later stages	Recovered	Saboo et al., 2012
11	2012	Tamil Nadu	42/M	Infection at the site of sutured scalp	Gentamycin	Recovered	Kumar, 2012
12	2013	Navi Mumbai	10/M	Septicemia	Ceftriaxone, Amikacin and Metronidazole	Fatal	Kar et al., 2013
13	2014	Orissa	19/M	UTI	Ciprofloxacin	Recovered	Swain et al., 2014
14	2015	South India	53/F	Septicemia	Imipenem, Ciprofloxacin, PTZ	Recovered	Madi et al., 2015
15	2016	Kerala	11month/M	Septicemia		Fatal	Kamjarakkal et al., 2016
16	2016	Kerala	2.5/M	Respiratory Distress, Hypotension, Shock		Fatal	
17	2016	Kerala	12/F			Recovered	
18	2016	Kerala	55/F	Catheter related blood stream infection	PTZ	Recovered	Balarama et al., 2016
19	2017	Kerala	73/M	UTI		Recovered	Vincent et al., 2017
20	2017	Madhya Pradesh	2/M	Chest abscess and ulceration	Ceftriaxone, Amikacin, Meropenem		Ahmed et al., 2017

Table P: Calculation of biomass composition using legacy data

Overall Composition

Component	% Dry Weight	Reference	Notes
Protein	71	(1)	Using *Neisseria* values as no clear literature found for *C.violaceum*.
RNA	30.3	(Herbert, 1961)	Exponential cells of c. violaceum in complex medium contains RNA % as 30.3.
DNA	13	(2)	
Phospholipids	11.4	(1)	Using neisseria values as no clear literature found for *C.violaceum.*
Peptidoglycan	0.1	(2)	
Lipopolysaccharide	7.6	(2, 3)	Average values between 7.61 and 7 %
PHB	38	(4)	98% PHB formed with the yield of 38% of dry weight with glucose as carbon source
Putrescine	0.4	(5)	*C. violaceum* values
Spermidine	0.004	(5)	*C. violaceum* values
Energy (mmol ATP/gDCW)	59.81	(6)	*E. coli* values

Component	% Dry Weight	Organism (Reference)
Protein	**41.33**	*N.meningitidis*
RNA	**17.64**	*C.violaceum*
DNA	**7.57**	*C.violaceum*
Phospholipids	**6.64**	*N.meningitidis*
Peptidoglycan	**0.06**	*C.violaceum*
Lipopolysaccharide	**4.42**	*C.violaceum*
PHB	**22.12**	*C.violaceum*
Putrescine	**0.231**	*C.violaceum*
Spermidine	**0.003**	*C.violaceum*
Total	**100**	

DNA Composition

Reference - Haselkorn R, Artur L, Bataus M, Batista S, Teno C: The complete genome sequence of *Chromobacterium violaceum* reveals remarkable and exploitable bacterial adaptability. *Proc Natl Acad Sci U S A* 2003, 100:11660–11665.

DNA	% Prevalence	MW (g/mol)	Relative Weight/mol	% (by weight)	**mmol/gDW**
dATP	17.6	487.151	85.74	17.75	**0.028**
dGTP	32.4	503.15	163.02	33.75	**0.051**
dTTP	17.6	478.136	84.15	17.42	**0.028**
dCTP	32.4	463.125	150.05	31.07	**0.051**
	Total		Sum of Rel Weight/mol	Total	
	100		482.96	100	

Explanation:

The 64.8% GC content is split evenly between dGTP and dCTP. MWs taken from ChEBI website.

The relative weights/mol are the % prevalances multipled by the molecular weights.

% by weight is calculated by divding the relative weights/mol by the sum of the relative

weights/mol.
mmol/gDW is calculated by:
(% by weight/100)*(X grams of DNA/gDW)*(1/molecular weight)*(1000 mmol/mol)

RNA Composition
Reference - ORF DNA sequences (proxy for RNA) for *C. violaceum* used from Haselkorn et. al 2004

RNA	% Prevalence	MW (g/mol)	Relative weight/mol	% (by weight)	mmol/gDW
ATP	18.00	503.15	90.57	18.24	**0.064**
GTP	32.00	519.149	166.13	33.46	**0.114**
UTP	18.00	480.108	86.42	17.41	**0.064**
CTP	32.00	479.124	153.32	30.88	**0.114**
	Total		Sum of Rel Weight/mol	Total	
	100		496.43	100	

Explanation
MWs taken from ChEBI website. The relative weights/mol are the % prevalances multipled by the molecular weights. % by weight is calculated by divding the relative weights/mol by the sum of the relative weights/mol.
mmol/gDW is calculated by:
(% by weight/100)*(X grams of RNA/gDW)*(1/molecular weight)*(1000 mmol/mol)

Protein Composition
Protein sequence for *C. violaceum* ATCC 12472 was downloaded from Uniprot Proteome ID - UP000001424

Amino Acid	Count	% Prevalence	MW (g/mol)	Relative Weight/mol	% (by weight)	mmol/gDW
Alanine (A)	174329	12.49	89.09	11.13	8.77	**0.407**
Arginine (R)	95825	6.87	175.212	12.03	9.48	**0.224**
Asparagine (N)	40011	2.87	132.12	3.79	2.98	**0.093**
Aspartic acid (D)	75976	5.44	132.1	7.19	5.67	**0.177**
Cysteine (C)	14379	1.03	121.16	1.25	0.98	**0.034**
Glutamate (E)	75054	5.38	146.12	7.86	6.19	**0.175**
Glutamine (Q)	61225	4.39	146.14	6.41	5.05	**0.143**
Glycine (G)	117225	8.40	75.06	6.31	4.97	**0.274**
Histidine (H)	30530	2.19	155.15	3.39	2.67	**0.071**
Isoleucine (I)	61630	4.42	131.17	5.79	4.56	**0.144**
Leucine (L)	160400	11.49	131.17	15.08	11.88	**0.374**
Lysine (K)	50092	3.59	146.19	5.25	4.13	**0.117**
Methionine (M)	34257	2.45	149.21	3.66	2.89	**0.080**
Phenylalanine (F)	48018	3.44	165.19	5.68	4.48	**0.112**
Proline (P)	69184	4.96	115.13	5.71	4.50	**0.161**
Serine (S)	78774	5.65	105.09	5.93	4.67	**0.184**

Threonine (T)	59258	4.25	119.19	5.06	3.99	**0.138**
Tryptophan (W)	20482	1.47	204.22	3.00	2.36	**0.048**
Tyrosine (Y)	34649	2.48	181.19	4.50	3.54	**0.081**
Valine (V)	94136	6.75	117.15	7.90	6.23	**0.220**
	Sum	Total		Sum of Rel Weight/mol	Total	
	1395434	100		126.93	100	

Explanation
MWs taken from ChEBI website. The relative weights/mol are the % prevalances multipled by the molecular weights. % by weight is calculated by divding the relative weights/mol by the sum of the relative weights/mol. mmol/gDW is calculated by:
(% by weight/100)*(X grams of protein/gDW)*(1/molecular weight)*(1000 mmol/mol)

Polyamine Composition
Reference - Busse J, Auling G: Polyamine Pattern as a Chemotaxonomic Marker within the Proteobacteria. Syst Appl Microbiol 1988, 11:1–8.

Polyamine	umol/gDW	mmol/gDW	MW (g/mol)	% dry weight
Putrescine	44.1	0.0441	90.2	**0.397782**
Spermidine	0.3	0.0003	148.32	**0.00445**
Cadaverine	7.3	0.0073	104.23	**0.076088**
1,3-diaminopropane	5.6	0.0056	74.15	**0.041524**
2 hydroxyputrescine	23.2	0.0232	104.18	**0.241698**
			Total	**0.761541**

Peptidoglycan Composition

Peptidoglycan	% dry weight	MW (g/mol)	mmol/gDW
n subunit	0.06	1983.21	**0.000293**
n-1 subunit	0.06	993.12	**0.000586**

Model Information			
Code	Name	Charged Formula	MW
cpd15665_c	Peptidoglycan polymer (n subunits)	$C_{80}H_{125}N_{16}O_{42}R$	1983.21
cpd15666_c	Peptidoglycan polymer (n-1 subunits)	$C_{40}H_{63}N_8O_{21}R$	993.12

Lipopolysaccharide Composition
Reference - Hase S, Reitschel ET: The chemical structure of the lipid A component of lipopolysaccharides from *Chromobacterium violaceum* NCTC 9694. Eur J Biochem 1977, 75:23–34.

Lipid A is the representative component of LPS in the E. coli model. We have done the same here.

New_metabolite	*C. violaceum* Lipid A	C91H165N4O33P2	
		MW (g/mol)	
C	91	12.01	1092.91
H	165	1.01	166.65
N	4	14.01	56.04
O	33	16	528
P	2	30.97	61.94
			1905.54
Lipopolysaccharide	% dry weight	MW (g/mol)	mmol/g DW
LPS	4.42	1905.54	**0.023215**

Fatty Acid Composition Part 1

Reference - Kämpfer P, Busse HJ, Scholz HC. Int J Syst Evol Microbiol 2009 and Young C-C, Arun a B, Lai W-A, Chen W-M, Chou J-H, Shen F-T, Rekha PD, Kämpfer P. Int J Syst Evol Microbiol 2008

	Fatty acid	C	H	O	MW (g/mol)	% Prevalence of Total FA in *C. violaceum*	% Over all Preva lence	Weighted MW (g/mol)	
Unsaturated fatty acid	16:1	16	29	0. 5	229.408	35.8	36.95	84.75529928	Palmitoleate
	18:1	18	33	0	249.462	15	15.48	38.61640867	omega-7-cis-octadecenoic acid
Saturated fatty acid	12:0	12	23	0	167.316	5.6	5.78	9.669448916	dodecanoic acid
	14:0	14	27	0	195.37	1.7	1.75	3.42754386	tetradecanoic acid or myrisitic acid
	16:0	16	31	0	223.424	23.9	24.66	55.1066419	hexadecanoic acid or palmitic acid
Cyclopropa ne fatty acid	17:0cyc	17	31	0	235.435	1.7	1.75	4.130438596	Cycloheptadecanoic acid
B-hydroxy FA (only LPS)	10:0-3OH	10	19	1	155.261	5.2	5.37	8.331859649	3-hydroxydecanoate
	12:0-2OH	12	23	1	183.315	3	3.10	5.675386997	2-hydroxydodecanoate
	12:0-3OH	12	23	1	183.315	5	5.16	9.458978328	3-hydroxydodecanoate
Sum						96.9	100	Average Fatty Acid MW	

Lipids	% Prevalance	Specific Lipids	% Prevalance	Overall % Prevalance	Number of C	Number of H	Number of O	Number of P	Number of N	Number of FA	MW (g/mol)	Relative Weight/mol	% by weight	mmol/g DW
Phospholipids	100	PE	76.6	76.60	1791	3001	432	50	50	2	34135.7	26147.9	73.42	**0.001427**
		PG	17.9	17.90	1841	3001	532	50	0	2	35635.8	6378.8	17.91	**0.000334**
		CLPN (DPG)	4.6	4.60	3532	6547	882	100	0	4	67107.4	3086.94	8.67	**8.57E-05**
		PA	0.9	0.90										
	Total		100	Total								Sum	Total	
	100			99.10								35613.7	100	

Fatty Acid Composition Part 2

Reference - Kämpfer P, Busse HJ, Scholz HC. Int J Syst Evol Microbiol 2009 and Young C-C, Arun a B, Lai W-A, Chen W-M, Chou J-H, Shen F-T, Rekha PD, Kämpfer P. Int J Syst Evol Microbiol 2008

Lipids	% Prevalence	Head Group	% Prevalence	MW (g/mol)	Number of Fatty Acids	Fatty Acid	% Prevalence	Overall % Prevalence	MW (g/mol)	Relative MW/mol	% by weight	mmol/gDW
Phospholipids	100	phosphatidylethanolamine (PE)	76.6	269.15	2	PE 12:0	6.69	5.12	603.78	30.94	4.11	**0.0045**
						PE 14:0	2.03	1.56	659.89	10.27	1.36	**0.0014**
						PE 16:0	28.55	21.87	716.00	156.61	20.79	**0.0193**
						PE 16:1	42.77	32.76	727.97	238.50	31.67	**0.0289**
						PE 18:1	17.92	13.73	768.07	105.44	14.00	**0.0121**
						PE 17:0cyc	2.03	1.56	740.02	11.51	1.53	**0.0014**
		phosphatidylglycerol (PG)	17.9	300.16	2	PG 12:0	6.69	1.20	634.79	7.60	1.01	**0.0011**
						PG 14:0	2.03	0.36	690.90	2.51	0.33	**0.0003**
						PG 16:0	28.55	5.11	747.01	38.18	5.07	**0.0045**
						PG 16:1	42.77	7.66	758.98	58.11	7.72	**0.0067**
						PG 18:1	17.92	3.21	799.08	25.63	3.40	**0.0028**

				PG 17:0cyc	2.03	0.36	771.03	2.80	0.37	**0.0003**
cardiolipin (CLPN)	4.6	508.22	4	CLPN 12:0	6.69	0.31	1177.48	3.62	0.48	**0.0003**
				CLPN 14:0	2.03	0.09	1289.70	1.20	0.16	**0.0001**
				CLPN 16:0	28.55	1.31	1401.92	18.41	2.44	**0.0012**
				CLPN 16:1	42.77	1.97	1425.85	28.05	3.72	**0.0017**
				CLPN 18:1	17.92	0.82	1506.07	12.42	1.65	**0.0007**
				CLPN 17:0cyc	2.03	0.09	1449.96	1.35	0.18	**0.0001**
	99.1			small % of other FAs	300.00			**Sum**		
						99.1		753.18		

Material A: SEED Draft model limitation

The SEED-derived *in silico C. violaceum* was unable to produce twenty six out of 74 biomass precursors as reported in literature (7, 8) with glucose as the sole carbon source. A total of 69 reactions (Table E in this file) were subsequently added in order to have a functional biomass equation to represent growth in the GSMM of *C. violaceum*. Seven reactions were added to lipopolysaccharide biosynthesis, 6 reactions were added to fatty acid metabolism and KDO2 lipid biosynthesis subsystems. Five reactions were added to four subsystems namely riboflavin metabolism, thiamine metabolism, folate biosynthesis and porphyrin metabolism. Purine metabolism, glyoxylate and dicarboxylate metabolism, nicotinate and nicotinamide metabolism needed four missing reactions in order to form biomass. Other subsystems that were missing reactions and were gap filled included terpenoid biosynthesis, biotin metabolism, fatty acid biosynthesis, phenylalanine, tyrosine and tryptophan biosynthesis, urea cycle and metabolism of amino groups, vitamin B6 metabolism, glycine, serine and threonine metabolism, TCA cycle, ubiquinone biosynthesis, glutathione metabolism, spermidine biosynthesis (urea cycle), glycolysis/ACP, aminosugar metabolism, lysine biosynthesis and peptidoglycan biosynthesis. Twenty two reactions were added to account for synthesis of the fatty acids and phosphoglycerides unique to *C. violaceum* in fatty acid biosynthesis and glycerolipid and glycerophospholipid metabolism subsystem in the model based on legacy data (9, 10). A total of 143 new reactions added with a prefix of “rDB” and 20 new metabolites were added with “mDB” prefix and the average confidence score for the model was 1.45. Although the genome of *C. violaceum* has no missing components in the biosynthetic pathways of all 20 amino acids and the purine/pyrimidine, the SEED reconstruction (http://www.theseed.org/models/) did not reflect these features. This shows that although automated draft reconstructions for metabolic models (11) are a great starting point, detailed manual curation is a necessity for complete reconstructions that can be

translated into predictive models that can simulate cell function accurately and maintain standards (12).

Pathways with gaps and missing reactions included lysine, phenylalanine, tyrosine and tryptophan biosynthesis and glycine, serine and threonine metabolism pathways. *C. violaceum* does not require any amino acids for its growth (8) (13). Many genes were not identified, annotated or mis-annotated. In case of lysine biosynthesis a lumped reaction had to be added for the conversion of L-aspartate semi-aldehyde to meso-2,6-diaminopimelate (rDB00050_c) in order to form lysine in the reconstruction. For arginine biosynthesis reaction (rDB00003_c) was added for formation of arginine (glutamate and 2acetamido-5-oxopentanoate to oxoglutarate and N-acetylornithine). Tryptophan metabolism in *C. violaceum* is unique as compared to other bacteria due to its oxidative conversion to natural bis-indole compound violacein (14–16) through a five gene violacein operon (17–19). In the SEED reconstruction the reaction that converts chorismate to prephenate (Chorismate mutase or prephenate dehydratase, CV_2355, *pheA*) was missing (Figure B in this file) although indirect evidence (20) supports its occurrence in the network for biosynthesis of phenylalanine, tyrosine and biomass formation. In case of glycine, serine threonine metabolism we added a reaction (rDB00029_c) for linking 1-amino-2-propanol to aminoacetone. Synthesis of variety of cofactors and vitamins were also complete with the exception of pantothenate and biotin biosynthesis. Vitamin B12 was represented by Vitamin B12 coenzyme adenosylcobalamin (adocbl_c) in the model and was synthesized by *i*DB858 and is also included in the biomass equation as suggested in literature to be important for cellular functioning (8).

Material B: *In silico* representation of metabolic genome features of *Chromobacterium violaceum*

In 2003, the *C. violaceum* ATCC 12472 genome sequencing project was executed by the Brazilian National Genome Sequencing Consortium that included 25 sequencing laboratories, 1 bioinformatics center, and 3 coordination laboratories spread across Brazil (21). Of the 4407 protein coding genes only 61.3% could be assigned a putative function whereas 21.6% were identified as conserved hypothetical proteins and 17.1% other hypothetical proteins.

On comparison with other sequenced organisms, *C. violaceum* has been reported to be most similar to (17.4%) *Ralstonia solanacearum,* a free living phytopathogen (21). Although phylogenetically similar to the serious human pathogen *N. meningitidis* serogroup A (9.75%), and that of the Clusters of Orthologous Groups (COG) of ribosomal structure, biogenesis and translation.(1), similarity of COGs of inorganic ion transporters to Ralstonia predicted *C. violaceum* to be free living rather than a commensal.

C. violaceum was proposed to be well adapted to glucose, nitrogen, phosphate and amino acid starvation and is resistant to toxic agents such as hydrogen peroxide, arsenic (22), UV radiation, oxidative damage due to presence of several ORFs that act in response to such stress like pho regulon, peptide utilization and heat shock related ORFs. Around 251 genes incorporated in the model had direct literature evidence. The model *i*DB858 was able to successfully predict the physiology of *C. violaceum* as per legacy data (Table 2).

Central Carbon Metabolism

The *in silico C. violaceum, i*DB858, was able to synthesize all the necessary amino acids for its survival and also showed ability to synthesize cyanide (23). As previously reported (24) during aerobic growth on glucose it was able to use glycolysis, tricarboxylic acid and glyoxylate cycle to produce cellular energy required for cell survival. The model was able to

utilize amino acids, lipids and acetonitrile as sole carbon sources (25). The latter was utilized by the presence of homologous nitrilase (CV_2097) that allowed utilization of nitriles compounds such as indole acetonitrile, benzonitrile, phenylacetonitrile as suggested in literature (26). All the genes required for nucleotide salvage pathway were accounted for in the model.

In glycolysis *pck, agp, crr, ascF, eutG, pdc* genes were absent but *ppc* gene, CV_0055 was present in addition to CV_2491. In TCA *pfo, mqo, pck* genes were absent. In PPP *kdgK, ghrB, gnd, gcd,* genes were absent. Absence of *talB* (transaldolase) was compensated by *talc* (CV_2247) transaldolase activity. In pyruvate metabolism *pck, mqo, pdc* (pruvate decarboxylase)*, aldA, hchA, ldhA, lldA* (EC 1.1.2.3, lactate dehydrogenase)*, lldD, poxB, pfo, yccX, eutD, maeA, mhpF* (EC 1.2.1.10, acetaldehyde-CoA dehydrogenase II, NAD-binding) genes were absent but CV_2491 and *dld* (CV_3027 – D-lactate dehydrogenase) were present and *adhE* (CV_1137) replaced *mhpF* function in the model.

All the respiratory complexes (Complex I to V) were present in the model as reported in literature. Electrons entered the respiratory chain through NADH dehydrogenase (EC 1.6.5.3, 14 genes *nuoA* to *nuoN*) or succinate dehydrogenase (EC 1.3.5.1, *sdhA* to *sdhD*) and were transferred to the cytochrome bc1 or cytochrome c reductase (EC 1.10.2.2, *petA, petB* and *petC*) complex through ubiquinones. The quinone system in the model was represented by ubiquinone Q-8 (27). Two types of terminal cytochrome oxidase (EC 1.9.3.1) were reported (7) in *C. violaceum*, SoxM was represented by *coxA* (CV_0600), *coxB* (CV_0599) and *coxC* (CV_0603) whereas FixN was represented by CV_1171, CV_1172, CV_1173 and CV_1174 based on homology. SoxM type (aa3 type) worked under normal aerobic conditions whereas the FixN oxidases were involved under micro-aerophillic conditions (28) that may allow colonization of oxygen-limited environments. Another terminal oxidase, cytochrome bd

oxidase was also reported in *C. violaceum* known to play a role in oxidative stress and to create electrochemical membrane gradient for energetic requirements (28). Cyanide formation in *C. violaceum* is a distinguishing feature among violacein producing bacteria. In general, cyanide binds with the respiratory electron chain molecule and inhibits respiration and kills the cells. Therefore, there must be an evolved respiratory system that is resistant to cyanide production as reported (29). *cioA* (CV_3658), a cyanide insensitive terminal cytochrome oxidase in the respiratory electron transport (30) and *cioB* (CV_3657) were genes present in *in silico C. violaceum* model homologous to the cytochrome bd (EC 1.10.3.14) oxidases and may belong to cyanide insensitive oxidases (CIO) as observed in *Pseudomonas aeruginosa* (31) suggesting terminal branching of the respiratory system in *C. violaceum* with one pathway resistant to cyanide inhibition (or azide, CO inhibition) while the other being sensitive (29). *C. violaceum* being a facultative anaerobe reactions involving nitrate (denitrification) or fumarate as terminal electron acceptors for growth under anaerobic conditions were present that convert glucose into acetic acid and formic acid under anaerobic condition (7, 32, 33).

Macromolecular Biosynthesis

The cell envelope of *C. violaceum* similar to *N. meningitidis* and *E. coli* consists of an outer membrane, a peptidoglycan and a cytoplasmic (inner) membrane. The outer membrane has an asymmetrical organization in which the outside layer is primarily composed of lipopolysaccharide (LPS) (2, 34) and proteins whereas the inside membrane contains phospholipids. Bacterial lipids in *C. violaceum* like in all gram negative bacteria including *S.marcescens, E. aerogenes* and *E. coli* contains phosphatidylethanolamine (PE) as the major phosphoglyceride, and lesser amounts of phosphatidylglycerol (PG), phosphatidic acid (PA) and diphosphatidylglycerol (cardiolipin, CL)(35). The overall phospholipid composition used

in the model was 76.6% PE, and 17.9% PG, 4.6% CL and 0.9% PA based on legacy data (9, 35). Metabolic reactions for lipids used for various cellular functions including triacyglycerol, phospholipids and lipopolysaccharides were also included in the model. The fatty-acid biosynthesis subsystem in the model was curated using the template from *E. coli*. All genes, except for a homolog of the *E. coli* β-hydroxyacyl-acyl carrier protein (ACP) dehydrase FabA and FabB (3-oxoacyl-[acyl-carrier-protein] synthase I, 2.3.1.41), were present in the model in addition to CV_2194, a hypothetical protein (enoyl-[acyl-carrier protein] reductase II, 1.3.1.9). Homologs to *E. coli* glycerol-3-phosphate acyltransferase (PlsB) were not found so absent in the model on the other hand PlsX homolog CV_3417 and CV_3688 homologous to PlsY were present in *i*DB858. The unique fatty acid compositions for *C. violaceum* containing cycloheptane rings (9, 10) were also added in the model. The glycerophospholipid and glycerolipid metabolism were modified accordingly using reactions from *E. coli* iAF1260 (6) and *Burkholderia* reconstructions (36) based on gene sequence similarity.

The lipopolysaccharide (LPS) and peptidoglycan of *C. violaceum* are known to be responsible for activating host immune cells and the induction of inflammatory cytokines during bacterial infection, resulting in septic shock (37). Two type III secretion systems (T3SSs) encoded in three gene clusters (*Chromobacterium* pathogenicity islands Cpi-1, Cpi-1a and Cpi-2) are thought to be one of the most important virulent factors (38, 39) and Cpi-1/-1a T3SS are involved in the formation of necrotic lesions in the liver (38). LPS consists of three parts: a lipid A part containing unique hydroxy fatty acid chains, a core oligosaccharide containing 3-deoxy-D-manno-octulosonate (KDO) and heptoses, and a specific polysaccharide (somatic O-antigen). *C. violaceum* lipid A has been reported to be structurally different compared to other *Enterobactericeae* like *Salmonella* and *E. coli* (40). In *C. violaceum* lipid A the phosphate groups are substituted each by a distinct, non-acylated,

amino sugar. The glycosidically linked phosphate group was shown to be substituted by a glucosaminyl residue. This area, therefore, contains the structure of a glucosaminyl-1 - phosphoryl-1-glucosaminide. The ester-linked phosphate group was found to be substituted by a 4-aminoarabinosyl residue. All genes involved in the biosynthesis of the lipid A of LPS are present in *i*DB858 including *lpxB* (CV_2209) and *lpxD* (CV_2206) except for *lpxM* (myristoyl-acyl carrier protein (ACP)-dependent acyltransferase) that is absent in all betaproteobacteria (41). These genes are known to be related to virulence and pathogenicity in *C. violaceum* (37). The specific polysaccharide or O-antigen has been reported to be composed of D-glycero-D-mannoheptose and D-fucosamine (42) along with galactose, glucose, glucosamine (3). Peptidoglycan degree of cross-linking and O-acetylation appeared to be associated with the genetic background of the strains and was found to be quite similar to *N. meningitidis*. The percentage of O-acetylation per disaccharide was on average 14.7% compared to 33% in *N. meningitidis* (43). Other studies show that peptidoglycan structures are recognized by the innate immune system (39). Several studies have shown that a direct correlation exists between the extent of O- acetylation and susceptibility to lysozyme-catalyzed hydrolysis of peptidoglycan to protect the bacterium from a host immune response (43). In our model genes including CV_4346 and CV_4349 were present that are known to be related to virulence and pathogenicity in the peptidoglycan biosynthesis subsystem. *C. violaceum* has strong ability to adapt to stress condition due to presence of different types of transporter proteins. 25% of extracellular proteins have been reported to be involved in transport or metabolism (44, 45).

Cyanide Formation

Cyanide is produced by *C. violaceum* (46) as a secondary metabolite that has application in pharmaceuticals industry to gold recovery from electronic scrap materials (22, 23, 47, 48).

Cyanide formation in *C. violaceum* is also used as a distinguishing feature among violacein producing bacteria. Culture conditions such as pH, temperature regulate cyanide production (49). ^{14}C studies have showed carbon atom of cyanide being derived from glycine (50). *In silico* model of *C. violaceum* was able to produce small amounts of cyanide without any additives in the media with either only glucose or succinate as carbon source and ammonium salts as nitrogen source. Glutamate on the other hand served as both carbon and nitrogen source with best cyanide yield (23) (Table 2). The reactions involved in utilization of cyanide to form β-cyanoalanine (49, 50) and β -cyano-α-amino butyric acid were added to the model along with other reactions shown in Table E in this file. There is no inhibition of cytochrome c oxidase with the level of cyanide produced (30).

Violacein biosynthesis

The main precursor metabolite for the synthesis of violacein is tryptophan (51). Violacein biosynthetic pathway has a five gene vioABCDE operon structure (17–19, 52, 53). The operon is reported to be negatively regulated by VioS (54) and it is positively regulated by the CviI/R quorum sensing system. The first step is the oxidative dimerization of two molecules of tryptophan to indole-3-pyruvic acid (IPA) imine catalyzed by flavoenzyme L-tryptophan oxidase, VioA (CV_3274) in presence of oxygen(16). IPA imine formed is converted to protodeoxyviolaceinic acid by VioB (CV_3273) and VioE (CV_3270). This is followed by the hydroxylation activity of VioD (CV_3271) and VioC (CV_3272) to form violacein and deoxyviolacein. In the reaction involving VioA, H_2O_2 is formed that doesn't inhibit the functioning of the VioA rather stimulates higher production of protodeoxyviolacein. The flavin dependent oxygenases VioC and VioD work in simultaneous manner. In the presence of VioC, oxygen and NADPH, deoxyviolaceinic acid is formed from protodeoxyviolaceinic acid that is converted to deoxyviolacein in a non-enzymatic pathway

in the presence of oxygen. If VioD acts on protodeoxyviolaceinic acid before VioC*,* in the presence of NADPH and molecular oxygen, then it forms protoviolaceinic acid. VioC synthesizes violaceinic acid which later gets converted into violacein by spontaneous oxidative decarboxylation. In the absence of VioC, VioD and NADPH, protodeoxyviolaceinic acid gets converted into prodeoxyviolacein in the presence of oxygen spontaneously. The reactions (Table F in this file) involved in the violacein biosynthesis as discussed above have been added to the model. The robustness analysis of different control reactions including oxygen uptake, NADPH demand, ATP demand and tryptophan demand were studied on two different objective functions biomass and violacein production as shown in Figure 3. The slope for different control reactions suggested that availability of the substrate amino acid tryptophan and the cofactor NADPH were the major bottlenecks in violacein biosynthesis pathway in *i*DB858 as suggested in literature.

Supplementary References

1. Baart, G. J. E., Zomer, B., de Haan, A., van der Pol, L. a, Beuvery, E. C., Tramper, J., and Martens, D. E. (2007) Modeling Neisseria meningitidis metabolism: from genome to metabolic fluxes. *Genome Biol.* **8**, R136
2. WHEAT, R. W., ROLLINS, E. L., LEATHERWOOD, J. M., and BARNES, R. L. (1963) Studies on the cell wall of Chromobacterium violaceum: the separation of lipopolysaccharide and mucopeptide by phenol extraction of whole cells. *J. Biol. Chem.* **238**, 26–9
3. Crumpton, M. J., and Davies, D. a. L. (1958) The isolation of d-fucosamine from the specific polysaccharide of Chromobacterium violaceum (NCTC 7917). *Biochem. J.* **70**, 729–736
4. Kolibachuk, D., Miller, A., and Dennis, D. (1999) Cloning , Molecular Analysis , and Expression of the Polyhydroxyalkanoic Acid Synthase (phaC) Gene from Chromobacterium violaceum Cloning , Molecular Analysis , and Expression of the Polyhydroxyalkanoic Acid Synthase (phaC) Gene. *Appl. Environ. Microbiol.* **65**, 3561–3565
5. Busse, J., and Auling, G. (1988) Polyamine Pattern as a Chemotaxonomic Marker within the Proteobacteria. *Syst. Appl. Microbiol.* **11**, 1–8
6. Feist, A. M., Henry, C. S., Reed, J. L., Krummenacker, M., Joyce, A. R., Karp, P. D., Broadbelt, L. J., Hatzimanikatis, V., and Palsson, B. Ø. (2007) A genome-scale metabolic reconstruction for Escherichia coli K-12 MG1655 that accounts for 1260 ORFs and thermodynamic information. *Mol. Syst. Biol.* 10.1038/msb4100155

7. Creczynski-pasa, T. B., and Antônio, R. V (2004) Energetic metabolism of Chromobacterium violaceum. *Genet. Mol. Res.* **3**, 162–166
8. Demoss, R. D., and Happel, M. E. (1959) Nutritional requirements of Chromobacterium violaceum. *J. Bacteriol.* **77**, 137–141
9. Kampfer, P., Busse, H.-J., and Scholz, H. C. (2009) Chromobacterium piscinae sp. nov. and Chromobacterium pseudoviolaceum sp. nov., from environmental samples. *Int. J. Syst. Evol. Microbiol.* **59**, 2486–2490
10. Young, C.-C., Arun, a B., Lai, W.-A., Chen, W.-M., Chou, J.-H., Shen, F.-T., Rekha, P. D., and Kämpfer, P. (2008) Chromobacterium aquaticum sp. nov., isolated from spring water samples. *Int. J. Syst. Evol. Microbiol.* **58**, 877–880
11. Xu, Z., Fang, X., Wood, T. K., and Huang, Z. J. (2013) A Systems-Level Approach for Investigating Pseudomonas aeruginosa Biofilm Formation. *PLoS One*. **8**, 1–14
12. Ravikrishnan, A., and Raman, K. (2015) Critical assessment of genome-scale metabolic networks: the need for a unified standard. *Brief. Bioinform.* **16**, 1057–1068
13. Henry, C. S., DeJongh, M., Best, A. a, Frybarger, P. M., Linsay, B., and Stevens, R. L. (2010) High-throughput generation, optimization and analysis of genome-scale metabolic models. *Nat. Biotechnol.* **28**, 977–982
14. Durán, N., Justo, G. Z., Durán, M., Brocchi, M., Cordi, L., Tasic, L., Castro, G. R., and Nakazato, G. (2016) Advances in Chromobacterium violaceum and properties of violacein-Its main secondary metabolite: A review. *Biotechnol. Adv.* **34**, 1030–1045
15. Duran, M., Alario A. F., D. N. (2010) Chromobacterium violaceum and Its Important Metabolites – review. *Folia Microbioogy*. **55**, 535–547
16. Füller, J. J., Röpke, R., Krausze, J., Rennhack, K. E., Daniel, N. P., Blankenfeldt, W., Schulz, S., Jahn, D., and Moser, J. (2016) Biosynthesis of Violacein, Structure and Function of l-Tryptophan Oxidase VioA from Chromobacterium violaceum. *J. Biol. Chem.* **291**, 20068–20084
17. August, P. R., Grossman, T. H., Minor, C., Draper, M. P., MacNeil, I. A., Pemberton, J. M., Call, K. M., Holt, D., and Osburne, M. S. (2000) Sequence analysis and functional characterization of the violacein biosynthetic pathway from Chromobacterium violaceum. *J. Mol. Microbiol. Biotechnol.* **2**, 513–9
18. Hoshino, T. (2011) Violacein and related tryptophan metabolites produced by Chromobacterium violaceum: Biosynthetic mechanism and pathway for construction of violacein core. *Appl. Microbiol. Biotechnol.* **91**, 1463–1475
19. Balibar, C. J., and Walsh, C. T. (2006) In Vitro Biosynthesis of Violacein from <scp>l</scp> -Tryptophan by the Enzymes VioA−E from *Chromobacterium violaceum* †. *Biochemistry*. **45**, 15444–15457
20. Ronau, J. a., Paul, L. N., Fuchs, J. E., Corn, I. R., Wagner, K. T., Liedl, K. R., Abu-Omar, M. M., and Das, C. (2013) An additional substrate binding site in a bacterial phenylalanine hydroxylase. *Eur. Biophys. J.* **42**, 691–708
21. Haselkorn, R., Artur, L., Bataus, M., Batista, S., and Teno, C. (2003) The complete genome sequence of Chromobacterium violaceum reveals remarkable and exploitable bacterial adaptability. *Proc. Natl. Acad. Sci. U. S. A.* **100**, 11660–11665
22. Carepo, M. S. P., De Azevedo, J. S. N., Porto, J. I. R., Bentes-Sousa, A. R., Da Silva Batista, J., Da Silva, A. L. C., and Schneider, M. P. C. (2004) Identification of Chromobacterium violaceum genes with potential biotechnological application in environmental detoxification. *Genet. Mol. Res.* **3**, 181–194
23. Michaels, R., and Corpe, W. A. (1965) CYANIDE FORMATION BY CHROMOBACTERIUM VIOLACEUM. *J. Bacteriol.* **89**, 106–12
24. Banerjee, D., Parmar, D., Bhattacharya, N., Ghanate, A. D., Panchagnula, V., and Raghunathan, A. (2017) A scalable metabolite supplementation strategy against

antibiotic resistant pathogen Chromobacterium violaceum induced by NAD+/NADH+ imbalance. *BMC Syst. Biol.* **11**, 51
25. Chapatwala, K. D., Richardson, J. D., Nawaz, M., and Wolfram, J. H. (1988) Isolation and identification of acetonitrile degrading bacteria. in *Annual Meeting American Society Microbiology*, p. 225
26. A. Acharya, A. J. D. (1997) Studies on utilization of acetonitrile by Rhodococcus erythropolis A10. *World J. Microbiol. Biotechnol.* **13**, 175–178
27. Whistance, G. R., Dillon, J. F., and Threlfall, D. R. (1969) The nature, intergeneric distribution and biosynthesis of isoprenoid quinones and phenols in gram-negative bacteria. *Biochem. J.* **111**, 461–472
28. Castresana, J. (2001) Comparative genomics and bioenergetics. *Biochim. Biophys. Acta - Bioenerg.* **1506**, 147–162
29. Niven, D. F., Collins, P. a, and Knowles, C. J. (1975) The respiratory system of Chromobacterium violaceum grown under conditions of high and low cyanide evolution. *J. Gen. Microbiol.* **90**, 271–285
30. Tay, S. B., Natarajan, G., Rahim, M. N. B. A., Tan, H. T., Chung, M. C. M., Ting, Y. P., and Yew, W. S. (2013) Enhancing gold recovery from electronic waste via lixiviant metabolic engineering in Chromobacterium violaceum. *Sci. Rep.* **3**, 2236
31. Zlosnik, J. E. A., Tavankar, G. R., Bundy, J. G., Mossialos, D., O'Toole, R., and Williams, H. D. (2006) Investigation of the physiological relationship between the cyanide-insensitive oxidase and cyanide production in Pseudomonas aeruginosa. *Microbiology*. **152**, 1407–1415
32. Vemuri, G. N., Altman, E., Sangurdekar, D. P., Khodursky, a B., and Eiteman, M. a (2006) Overflow Metabolism in Escherichia coli during Steady-State Growth : Transcriptional Regulation and Effect of the Redox Ratio Overflow Metabolism in Escherichia coli during Steady-State Growth : Transcriptional Regulation and Effect of the Redox Ratio †. *Appl. Environ. Microbiol.* **72**, 3653–3661
33. Vemuri, G. N., Eiteman, M. A., McEwen, J. E., Olsson, L., and Nielsen, J. (2007) Increasing NADH oxidation reduces overflow metabolism in Saccharomyces cerevisiae. *Proc. Natl. Acad. Sci. U. S. A.* **104**, 2402–7
34. Whiteside, T. L., and Corpe, W. A. (1969) Effect of enzymes on the composition and structure of Chromobacterium violaceum cell envelopes. *J. Bacteriol.* **97**, 1449–59
35. Randle, C. L., Albro, P. W., and Dittmer, J. C. (1969) The phosphoglyceride composition of gram-negative bacteria and the changes in composition during growth. *Biochim. Biophys. Acta - Lipids Lipid Metab.* **187**, 214–220
36. Bartell, J. A., Yen, P., Varga, J. J., Goldberg, J. B., and Papin, J. A. (2014) Comparative Metabolic Systems Analysis of Pathogenic Burkholderia. *J. Bacteriol.* **196**, 210–226
37. Ghosh, R., Tiwary, B. K., Kumar, A., and Chakraborty, R. (2014) Guava leaf extract inhibits quorum-sensing and Chromobacterium violaceum induced lysis of human hepatoma cells: whole transcriptome analysis reveals differential gene expression. *PLoS One*. **9**, e107703
38. Miki, T., Iguchi, M., Akiba, K., Hosono, M., Sobue, T., Danbara, H., and Okada, N. (2010) Chromobacterium pathogenicity island 1 type III secretion system is a major virulence determinant for Chromobacterium violaceum-induced cell death in hepatocytes. *Mol. Microbiol.* **77**, no-no
39. Brito, C. F. A. de, Carvalho, C. B., Santos, F., Gazzinelli, R. T., Oliveira, S. C., Azevedo, V., and Teixeira, S. M. R. (2004) Chromobacterium violaceum genome: molecular mechanisms associated with pathogenicity. *Genet. Mol. Res.* **3**, 148–61
40. Hase, S., and Reitschel, E. T. (1977) The chemical structure of the lipid A component

of lipopolysaccharides from Chromobacterium violaceum NCTC 9694. *Eur. J. Biochem.* **75**, 23–34
41. Opiyo, S. O., Pardy, R. L., Moriyama, H., and Moriyama, E. N. (2010) Evolution of the Kdo2-lipid A biosynthesis in bacteria. *BMC Evol. Biol.* **10**, 362
42. Maclennan, a P., and Davies, D. a (1957) The isolation of D-glycero-D-galactoheptose and other sugar components from the specific polysaccharide of Chromobacterium violaceum (BN). *Biochem. J.* **66**, 562–567
43. Weadge, J. T., Pfeffer, J. M., and Clarke, A. J. (2005) Identification of a new family of enzymes with potential O-acetylpeptidoglycan esterase activity in both Gram-positive and Gram-negative bacteria. *BMC Microbiol.* **5**, 49
44. Ciprandi, A., Da Silva, W. M., Santos, A. V., De Castro Pimenta, A. M., Carepo, M. S. P., Schneider, M. P. C., Azevedo, V., and Silva, A. (2013) Chromobacterium violaceum: Important insights for virulence and biotechnological potential by exoproteomic studies. *Curr. Microbiol.* **67**, 100–106
45. Grangeiro, T. B., Jorge, D. M. D. M., Bezerra, W. M., Vasconcelos, a. T. R., and Simpson, a. J. G. (2004) Transport genes of Chromobacterium violaceum: An overview. *Genet. Mol. Res.* **3**, 117–133
46. Sneath, P. H. A., Singh, R. B., Whelan, J. P. F., and Edwards, D. (1953) FATAL INFECTION BY CHROMOBACTERIUM VIOLACEUM. *Lancet.* **262**, 276–277
47. Campbell, S. C., Olson, G. J., Clark, T. R., and McFeters, G. (2001) Biogenic production of cyanide and its application to gold recovery. *J. Ind. Microbiol. Biotechnol.* **26**, 134–139
48. Natarajan, G., and Ting, Y. P. (2014) Pretreatment of e-waste and mutation of alkali-tolerant cyanogenic bacteria promote gold biorecovery. *Bioresour. Technol.* **152**, 80–85
49. Macadam, A. M., and Knowles, C. J. (1984) Purification and Properties of Beta-cyano-L-alanine Synthase from the Cyanide-producing Bacterium, Chromobacterium Violaceum. *Biochim. Biophys. Acta.* **786**, 123–132
50. Brysk, M. M., Corpe, W. a., and Hankes, L. V. (1969) β-Cyanoalanine Formation by Chromobacterium violaceum. *J. Bacteriol.* **97**, 322–327
51. Demoss, R. D., and Evans, N. R. (1959) Physiological aspects of violacein biosynthesis in nonproliferating cells. *J. Bacteriol.* **78**, 583–588
52. Sánchez, C., Braña, A. F., Méndez, C., and Salas, J. a. (2006) Reevaluation of the violacein biosynthetic pathway and its relationship to indolocarbazole biosynthesis. *ChemBioChem.* **7**, 1231–1240
53. Shinoda, K., Hasegawa, T., Sato, H., Shinozaki, M., Kuramoto, H., Takamiya, Y., Sato, T., Nikaidou, N., Watanabe, T., and Hoshino, T. (2007) Biosynthesis of violacein: a genuine intermediate, protoviolaceinic acid, produced by VioABDE, and insight into VioC function. *Chem. Commun. (Camb).* 10.1039/b705358d
54. Devescovi, G., Kojic, M., Covaceuszach, S., Cámara, M., Williams, P., Bertani, I., Subramoni, S., and Venturi, V. (2017) Negative Regulation of Violacein Biosynthesis in Chromobacterium violaceum. *Front. Microbiol.* **8**, 1–11
